# Harmful incidents following gynaecological ambulatory surgery: A scoping review

**DOI:** 10.1016/j.ijnsa.2026.100487

**Published:** 2026-01-07

**Authors:** Cathrine Ween Thoen, Malin Knutsen Glette, Siri Wiig, Kim Christian Danielsson, Signe Berit Bentsen

**Affiliations:** a Department of health and caring sciences, Faculty of Health and Social Sciences, Western Norway University of Applied Sciences, Bergen, Norway; b Department of Anaesthesia and Intensive Care, Haukeland University Hospital, Bergen, Norway; c SHARE – Centre for Resilience in Healthcare, Faculty of Health Sciences, University of Stavanger, Stavanger, Norway; d Department of Obstetrics and Gynaecology, Haukeland University Hospital, Bergen, Norway; e Department of Clinical Science, University of Bergen Faculty of Medicine and Dentistry, Bergen, Norway; f Division of Emergencies and Critical Care, Department of Operating Services, Oslo University Hospital, Oslo, Norway

**Keywords:** Ambulatory surgery nursing, Ambulatory surgical procedures, Day surgery, Gynecology, Patient Safety, Perioperative Nursing, Review

## Abstract

**Aim:**

To map the types and frequencies of harmful incidents identified in research to occur following gynaecological ambulatory surgery.

**Design and methods:**

A scoping review was conducted using the JBI Manual for Evidence Synthesis.

**Data Sources:**

MEDLINE, Embase, CINAHL, Cochrane Library, Scopus and Web of Science was searched for records published from inception to February of 2025.

**Results:**

Forty-one studies were included. Most of the harmful incidents identified to have occurred following gynaecological ambulatory surgery were mapped as physiological harm, including postoperative infections, bleeding and pain. The only harmful incident pertaining to other dimensions of harm was anxiety, which was mapped as psychological harm.

**Conclusion:**

In this scoping review, all except one of the identified and mapped harmful incidents resulted from the physiological impact and tissue damage of undergoing surgical treatment. Anxiety was the only psychological harm identified to have occurred following gynaecological ambulatory surgery. No social harmful incidents were identified from the included studies.

**Protocol Registration:**

The Open Science Framework; https://doi.org/10.17605/OSF.IO/VD6CB.

**What is already known**

• Although gynaecological ambulatory surgery is considered safe, harmful incidents do occur during the postoperative recovery at home.
• Efforts to improve clinical practice should be based on current knowledge, but no review of harmful incidents occurring following gynaecological ambulatory surgery was found.

Alt-text: Unlabelled box dummy alt text

**What this paper adds**

• In this scoping review, harmful incidents occurring after discharge from gynaecological ambulatory surgery were identified.
• Mapping of data from the included studies revealed that most of the identified harmful incidents pertained to physiological harm, while psychological and social aspects of harm were rarely, if ever, reported.
• Findings from this study can inform future research and be used by healthcare professionals to improve patient safety in gynaecological ambulatory surgery practice.

Alt-text: Unlabelled box dummy alt text

## 1 Background

Ambulatory surgery is elective surgical treatment without an overnight stay in hospital (World Health Organization et al., 2007). The development and expansion of ambulatory surgery practice over the past decades has been encouraged by incentives related to increased efficiency and less use of healthcare resources (OECD, 2023). Simultaneously, advancements in minimally invasive surgical techniques and anaesthesiology have maintained the safety of ambulatory surgery (Laaboub et al., 2020; OECD, 2023). Thus, gynaecological surgeries such as hysterectomy, myomectomy, pelvic reconstructive surgery and laparoscopic surgery with or without robotic assistance are considered safe as ambulatory surgery treatment (M. L. Fowler et al., 2022; Madden et al., 2019; Robison et al., 2022).

Despite the advances to ensure that gynaecological ambulatory surgery is as safe, if not safer than in-patient treatment, patient safety incidents continue to be of critical concern due to the occurrence of harmful incidents following discharge (Ellinides et al., 2022; Emery et al., 2024; M. L. Fowler et al., 2022). Harmful incidents are classified as patient safety incidents that result in patient harm, encompassing both preventable harmful incidents (adverse events) and non-preventable harmful incidents (adverse reactions) (World Health Organization, 2020). In context of patient safety, the World Health Organization (WHO) (2010) refers to harm as impairment of body structure or function and harmful effects there from. Harm can be physiological, psychological or social, including injury, disease, suffering, disability that restricts participation in society and death (World Health Organization, 2010). After gynaecological ambulatory surgery, harmful incidents such as bleeding, pain, infections, wound problems and incidents related to the gastrointestinal, urinary and cardiovascular systems have been reported to occur and result in readmissions and reoperations (Matern et al., 2020; Robison et al., 2022; Zillioux et al., 2021). Furthermore, syntheses of evidence from qualitative studies on ambulatory surgery treatments within various surgical specialties have found that patients experience incidents such as reduced ability to participate in social activities and feelings of sadness, anxiety and abandonment following discharge (Sig et al., 2025; Thoen et al., 2023). Thus, harmful incidents not only increase costs and the use of healthcare resources, but also affect treatment outcomes and the quality of recovery. Therefore, it is essential to maintain a continued focus on patient safety to ensure that the risk of unnecessary harm associated with gynaecological ambulatory surgery is reduced to an acceptable minimum (World Health Organization, 2010).

To improve patient safety in outpatient settings such as ambulatory surgery practice, the harmful incidents occurring should be addressed (Levine et al., 2024). However, efforts to improve clinical practice should be based on knowledge of the realities and challenges of these harmful incidents (Dixon-Woods, 2010). From a preliminary search in MEDLINE, Embase, Cochrane, CINAHL, Scopus and Prospero, and to the best of our knowledge, there are no previous or ongoing reviews on harmful incidents after gynaecological ambulatory surgery. Thus, the aim of this scoping review was to map the types and frequencies of harmful incidents identified in research to occur following gynaecological ambulatory surgery.

## 2 Methods

This study was conducted using the chapter on Scoping Reviews in the JBI Manual for Evidence Synthesis (Peters et al., 2024). The protocol was published in Open Science Framework in May of 2023; https://doi.org/10.17605/OSF.IO/VD6CB. The manuscript has been reported according to the Preferred Reporting Items for Systematic Reviews and Meta-Analyses Extension for Scoping Reviews (PRISMA-ScR) checklist (Tricco et al., 2018) (Supplementary material 1).

### 2.1 Eligibility criteria

Eligible studies were published and peer-reviewed qualitative, quantitative and mixed-method empirical studies available in full text. No limits were applied to publication period or language. The participants were adult patients (≥ 18 years) undergoing gynaecological ambulatory surgery. Relevant outcomes were data on all harmful incidents reported to have occurred following the ambulatory surgery treatment. There were no restrictions regarding when the harmful incidents occurred following surgery, nor who reported them, such as patients, healthcare professionals or the researchers. This scoping review focused on surgically complex treatments. Thus, less extensive procedures such as hysteroscopies, colposcopies and interventions for urinary incontinence were excluded, except when performed as concurrent treatment. Studies focusing on drugs, anaesthesia, medical devices, reviews, opinion papers, dissertations, grey literature, white papers, books, book chapters, case studies, abstracts, protocols, editorials and commentaries were excluded.

### 2.2 Search strategy

The systematic literature search was conducted by an academic librarian (GTJ), in accordance with the stages recommended by JBI (Peters et al., 2024). The PCC elements of this scoping review, Population: adult patients (≥18 years), Concept: harmful incidents and Context: following discharge from gynaecological ambulatory surgery, were used to identify search terms for the initial limited search conducted in databases MEDLINE and CINAHL. The titles and abstracts of records retrieved from this initial search were reviewed and the search strategy was developed from text words and index terms identified in relevant records (GTJ, CWT). The final search strategy was peer-reviewed by a second academic librarian (McGowan et al., 2016). The systematic literature search was originally conducted in May of 2023 and updated in February of 2025 in the databases MEDLINE (through Ovid), Embase (through Ovid), CINAHL (through EBSCOhost), Cochrane Library, Scopus and Web of Science. Documentation of the systematic literature search is provided in Supplementary material 2. The reference lists of the included studies were manually screened for additional eligible sources (backward chaining) (CWT) (Peters et al., 2024).

### 2.3 Study selection

**Fig. 1:**
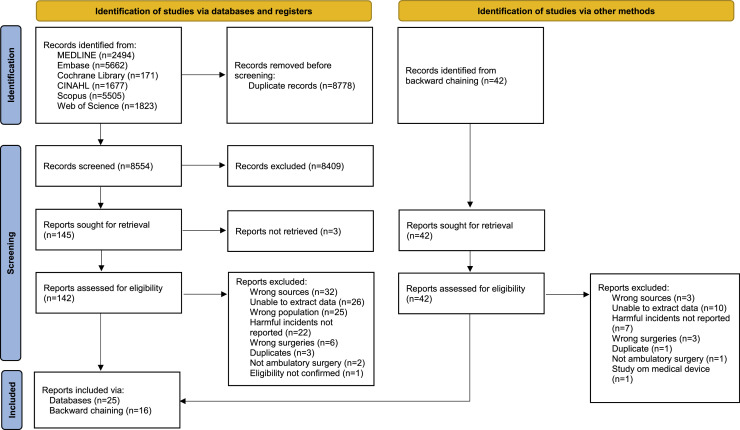
PRISMA flow diagram. Notes: Adapted from Page et al. (2021). This work is licensed under CC BY 4.0.

A total of 17,332 records were identified from the systematic literature search and uploaded to the EndNote reference management tool (version 20), where 8773 duplicates were removed (Akers, 2017; Bramer et al., 2016). Five additional duplicates were removed in the review management software DistillerSR, where the remaining records were transferred for study selection and data extraction. Throughout the subsequent process of study selection, the involved reviewers were blinded to each other’s decisions of inclusion or exclusion and conflicts of eligibility were resolved by consensus (Peters et al., 2024). Three reviewers tested the selection strategy for screening of study titles and abstracts by use of the eligibility criteria and an accompanying elaboration document (CWT, MKG, SBB). The three reviewers’ decisions of inclusion or exclusion were unanimous for 19 of the 25 studies that were randomly selected for the test (76 %), which according to JBI is a sufficiently high level of agreement to start screening (Peters et al., 2024). The studies with conflicting decisions were discussed, from which it was established that less extensive surgical procedures were to be excluded. The three reviewers then went on to complete the screening of remaining titles and abstracts, each study being assessed by two reviewers. Following screening, the number of potentially relevant studies was significantly reduced (Fig. 1). Thus, only two of the reviewers (CWT, SBB) went on to conduct the full text assessment and determine the final inclusion of studies in the scoping review. A list of the studies that were excluded based on full-text assessment and reasons for exclusion is provided in Supplementary material 3. One study was excluded because the types of gynaecological treatments included were not reported and the corresponding author was unable to provide additional information to verify eligibility (Bruun et al., 2023).

### 2.4 Data extraction

The data extraction form was developed in accordance with JBI’s manual and piloted on three studies by two reviewers (CWT, SBB) (Peters et al., 2024). From piloting the form, the following adjustments were made: The item ‘Objective(s) of the study’ was included in the section ‘Methodology’, the items ‘Methods of surgery’ and ‘Time of data collection’ was added to the section ‘Participants’, and the two items for types and occurrences of harmful incidents under ‘Concept’ were merged to avoid repetition (Supplementary material 4). To ensure that all relevant data were extracted from the included studies, the results and discussions as well as relevant tables and supplementary materials were reviewed. For the studies reported in English, two reviewers (CWT, SBB) separately extracted data from each study. Reviewer CWT then assessed both reviewers completed forms. If discrepancies were found, the two reviewers (CWT, SBB) discussed for clarification. Four of the included studies were reported in Spanish or French (Benoit et al., 2022; Calle et al., 2011; Guerrero-Machado et al., 2021; Rambeaud et al., 2017). For these studies, the quality of data translation and extraction was ensured by contributor MAHM, who is fluent in both languages and knowledgeable of the methods and concepts of this scoping review.

### 2.5 Analysis

Findings on study characteristics and the surgical treatments are presented in text, tables and figures. By deductive approach, all of the identified harmful incidents are mapped into main and subcategories under three dimensions of harm based on WHO’s *Conceptual Framework for the International Classification for Patient Safety* (2010); 1) physiological harm, 2) psychological harm and 3) social harm. In addition, the main categories of harmful incidents are mapped to provide an overview of the surgical treatments following which they occurred.

## 3 Results

### 3.1 Study characteristics

A total of 41 studies were included in this scoping review. The study selection process is presented in a PRISMA flow diagram (Fig. 1). Each included study is assigned an identification number (study ID) in Table 1. The studies were conducted in eight different Western and developed countries: The United States of America (26/41), Canada (5/41), Colombia (3/41), Denmark (2/41), France (2/41), the United Kingdom (1/41), Italy (1/41) and Spain (1/41). A total of 34,269 participants were included in the studies, with sample sizes ranging from 10 to 11,334. All the studies were quantitative, of which 11 had prospective designs (study IDs 5, 6, 11, 13–15, 25, 26, 31, 37, 38). The analysed data had been retrieved between January of 1991 to October of 2022, mostly from medical records and registries. The data was collected from the patients between 2 weeks to 12 months postoperatively, most commonly for approximately 30 days (19/41) (Table 1). Five studies did not report the follow-up time for data collection (study IDs 9, 12, 21, 25, 32). More details on study characteristics are provided in Table 1. The samples in two of the studies are likely to overlap (study IDs 27 and 30). Therefore, only the number of incidents extracted from Philp et al. (study ID 30) are included in counts of the harmful incidents that were reported in both studies, as these data were collected more recently and over a longer period.

**Table 1:** Study identification numbers (study ID), study characteristics and outcomes relevant to the scoping review.

**STUDY ID**	**FIRST AUTHOR (YEAR), COUNTRY**	**STUDY SETTING, SAMPLE SIZE AND DATA COLLECTION FOLLOW-UP TIME**	**STUDY OBJECTIVES**	**OUTCOMES**
1	Bazzurini et al. (2022), Italy	Setting: One national referral cancer centre. Sample size: 138. Data collection follow-up time: Up to 60 days.	To develop a same-day discharge setting for laparoscopic treatment of adnexal disease.	Adverse events.
2	Behbehani et al. (2020), USA	Setting: One tertiary academic care centre. Sample size: 441. Data collection follow-up time: 2 weeks.	To determine factors that predict urinary retention after same-day discharge minimally invasive hysterectomy.	Patient reported symptoms of urinary tract infection.
3	Benoit et al. (2022), France	Setting: Three hospitals. Sample size: 153. Data collection follow-up time: 1 month.	To assess the feasibility and safety of total hysterectomy by laparoscopic approach in outpatient surgery.	Factors associated with readmission and reoperation.
4	Delgado et al. (2021), USA	Setting: One academic-affiliated community hospital. Sample size: 176. Data collection follow-up time: 6 weeks.	To identify incidents of and risk factors for urinary retention after outpatient hysterectomy.	Data on urinary retention, pain and other symptoms.
5	Duffy et al. (2005), United Kingdom	Setting: Day surgery and outpatient units in three hospitals. Sample size: 22. Data collection follow-up time: 3 months.	To compare patient satisfaction, discomfort, procedure time, success rate and adverse events of hysteroscopic versus laparoscopic sterilisation.	Patient discomfort and postoperative adverse events.
6	Gale et al. (2018), Canada	Setting: One tertiary academic referral centre. Sample size: 44. Data collection follow-up time: 6 months.	To evaluate the feasibility of same-day discharge after laparoscopic hysterectomy, not excluding patients with complex surgical pathology and medical comorbidities.	Pain level, nausea and constipation.
7	Groth et al. (2022), Denmark	Setting: One department of gynaecology and obstetrics. Sample size: 728. Data collection follow-up time: 30 days.	To examine the feasibility of same-day discharge after pelvic organ prolapse surgery and investigate the cause of hospital contact after discharge.	Reasons for patient initiated contact and complications confirmed by gynaecologist.
8	Guerrero-Machado et al. (2021), Colombia	Setting: One hospital. Sample size: 67. Data collection follow-up time: Up to 22 days.	To describe the safety of discharge within 12 h after laparoscopic hysterectomy for benign uterine disease.	Safety variables such as complications.
9	Gunnala et al. (2016), USA	Setting: One study centre. Sample size: 207. Data collection follow-up time: Not reported.	To assess surgical outcomes and evaluate feasibility of same-day discharge after robot assisted myomectomy for myomas ≥9 cm.	Major adverse outcomes such as postoperative infection or abscess formation.
10	Lee et al. (2014), USA	Setting: One institution. Sample size: 157. Data collection follow-up time: 30 days.	To evaluate the feasibility and safety of same-day discharge after robot assisted hysterectomy with or without other procedures for benign and malignant conditions.	Reasons for postoperative health care visits/evaluations and readmissions.
11	Liu et al. (2019), USA	Setting: One ambulatory clinic at a tertiary institution. Sample size: 23. Data collection follow-up time: 6 weeks.	To determine safety and feasibility of same-day discharge in patients undergoing vaginal hysterectomy with pelvic floor reconstruction.	Postoperative complications.
12	Lloyd et al. (2018), USA	Setting: One tertiary care referral centre. Sample size: 10. Data collection follow-up time: Not reported.	To assess short term safety and patient satisfaction with same-day discharge after robotic assisted pelvic floor reconstructive procedures.	Postoperative outcomes.
13	Maheux-Lacroix et al. (2015), Canada	Setting: One tertiary hospital centre. Sample size: 128. Data collection follow-up time: 3 months.	To estimate feasibility and safety of total laparoscopic hysterectomy as an outpatient procedure for benign conditions.	Complications.
14	Mathew et al. (2022), USA	Setting: One tertiary referral centre. Sample size: 29. Data collection follow-up time: 4 weeks.	To asses patient preference and satisfaction with same-day discharge following transvaginal and minimally invasive apical pelvic organ prolapse repair surgery during the COVID-19 pandemic.	Patients' postoperative perspectives and complications.
15	Minig et al. (2015), Spain	Setting: One comprehensive cancer centre. Sample size: 24. Data collection follow-up time: 30 days.	To evaluate the feasibility and safety of multimodal perioperative care after laparoscopic hysterectomy.	Postoperative complications.
16	Moss et al. (2022), USA	Setting: One tertiary university-affiliated institution. Sample size: 142. Data collection follow-up time: 30 days.	To compare short term outcomes of same-day discharge with planned admission in patients undergoing apical pelvic organ prolapse repair.	Complications after surgery.
17	Penner et al. (2015), USA	Setting: Two study centres. Sample size: 118. Data collection follow-up time: 6 weeks.	To evaluate feasibility and safety of same-day discharge minimally invasive comprehensive surgical staging for endometrial and cervical cancer. (Main surgical treatment: Hysterectomy.)	Postoperative complications.
18	Raju et al. (2022), USA	Setting: A multicentre nationwide cohort. Sample size: 362. Data collection follow-up time: 30 days.	To investigate trends and outcomes of ambulatory minimally invasive sacrocolpopexy (pelvic organ prolapse surgery).	Postoperative outcomes.
19	Ross et al. (2022), USA	Setting: One tertiary referral centre. Sample size: 59. Data collection follow-up time: 30 days.	To compare the rates of urinary tract infection after apical pelvic organ prolapse surgery between patients undergoing same-day discharge and overnight recovery.	Postoperative variables such as postoperative urinary tract infections.
20	Tannus et al. (2022), USA	Setting: One centre at a tertiary academic hospital. Sample size: 618. Data collection follow-up time: 6 weeks.	To investigate the feasibility and predictive factors for same-day discharge after robot assisted hysterectomy for benign indications.	Postoperative variables.
21	Thomas et al. (2010), USA	Setting: One centre for assisted reproduction. Sample size: 189. Data collection follow-up time: Not reported.	To evaluate the efficacy and safety of mini laparotomy myomectomy in an ambulatory setting.	Postoperative complications.
22	Wield et al. (2022), USA	Setting: One centre at a women’s hospital. Sample size: 775. Data collection follow-up time: 30 days.	To determine feasibility and safety of same-day discharge after minimally invasive hysterectomy in gynaecologic oncology.	Postoperative complications.
23	Zhang et al. (2022), USA	Setting: Two university-affiliated hospitals. Sample size: 132. Data collection follow-up time: 30 days.	To assess the safety of same-day discharge following robot assisted endometrial cancer staging and identify risk factors for postoperative admission. (Main surgical treatment: Hysterectomy.)	Reasons for emergency department visits or admissions after discharge.
24	Berger et al. (2020), USA	Setting: A care organisation of 4.5 million members. Sample size: 5506. Data collection follow-up time: 30 days.	To compare the effect of same-day discharge on readmission risk after minimally invasive pelvic reconstructive surgery.	Reasons for hospital readmission and emergency department visits.
25	Calle et al. (2011), Colombia	Setting: Gynaecological endoscopy unit of one clinic. Sample size: 297. Data collection follow-up time: Not reported for all data. Pain assessment: 12 days.	To present the experience observed in a cohort of patients undergoing ambulatory total laparoscopic hysterectomy.	Postoperative outcomes, including complications, nausea and vomiting and pain.
26	Christiansen et al. (2019), Denmark	Setting: A regional teaching hospital. Sample size: 76. Data collection follow-up time: 28 days.	To investigate whether outpatient total laparoscopic hysterectomy could be performed as a routine without compromising patient satisfaction.	Complications.
27	Gien et al. (2011), Canada	Setting: One tertiary care academic teaching hospital. Sample size: 147. Data collection follow-up time: 3 weeks.	To evaluate feasibility of same-day discharge after laparoscopic gynaecologic oncology surgery. (Main surgical treatment: Hysterectomy or trachelectomy.)	Reasons for hospital admission.
28	Melamed et al. (2016), USA	Setting: Urban tertiary care centre at a teaching hospital. Sample size: 295. Data collection follow-up time: 30 days.	To investigate complications after same-day discharge laparoscopic hysterectomy for endometrial cancer and intraepithelial neoplasia.	Postoperative complications that occurred after surgery.
29	Perron-Burdick et al. (2011), USA	Setting: Data from care system register. Sample size: 527. Data collection follow-up time: 12 months.	To estimate readmission rates and emergency care use by patients discharged home the same day after laparoscopic hysterectomy.	Reasons for readmissions and urgent clinic or emergency department visits.
30	Philp et al. (2017), Canada	Setting: One institution. Sample size: 75. Data collection follow-up time: 30 days.	To evaluate the safety and feasibility of same-day discharge after laparoscopic radical hysterectomy for cervix cancer.	Reasons for unplanned health care encounters, readmissions and reoperations.
31	Rambeaud et al. (2017), France	Setting: One centre at a university hospital. Sample size: 13. Data collection follow-up time: 2 months.	To evaluate the feasibility of outpatient laparoscopic sacrocolpopexy (pelvic organ prolapse surgery).	Reasons for consultation with a doctor/ emergency department following surgery and postoperative pain.
32	Rendón et al. (2016), Colombia	Setting: One cancer institution. Sample size: 31. Data collection follow-up time: Not reported.	To report the feasibility of outpatient laparoscopic radical hysterectomy in patients with early-stage cervical cancer.	Postoperative complications and pain.
33	Rettenmaier et al. (2012), USA	Setting: A tertiary institution. Sample size: 21. Data collection follow-up time: 23 days.	To evaluate same-day discharge in clinical stage I endometrial cancer patients treated with total laparoscopic hysterectomy, salpingo-oophorectomy and pelvic lymph node dissection.	Postoperative complications.
34	Rivard et al. (2015), USA	Setting: A university hospital. Sample size: 90. Data collection follow-up time: 30 days.	To determine factors for safe outpatient robot assisted minimally invasive gynaecologic oncology surgery. (Main surgical treatment: Hysterectomy, salpingo-oophorectomy or trachelectomy.)	Postoperative complications.
35	Romanova et al. (2020), USA	Setting: One tertiary care institution at a women’s hospital. Sample size: 258. Data collection follow-up time: 30 days.	To evaluate unanticipated healthcare encounters after same-day discharge major pelvic organ prolapse surgery and assess postoperative complications and severity.	Reasons for unplanned healthcare encounters after surgery.
36	Schiff et al. (2019), USA	Setting: The American College of Surgeons National Surgical Quality Improvement Program database. Sample size: 6000. Data collection follow-up time: 30 days.	To assess the effect of length of hospital stay on 30-day postoperative outcomes after minimally invasive hysterectomy.	Surgical site infections (superficial, incisional, and/or organ space) and urinary tract infections.
37	Stovall et al. (1992), USA	Setting: A regional medical centre. Sample size: 35. Data collection follow-up time: 6 weeks.	To determine the feasibility and safety of outpatient vaginal hysterectomy and patient acceptance of the procedure.	Safety variables such as reasons for readmission.
38	Casas-Puig et al. (2024), USA	Setting: One tertiary care centre. Sample size: 76. Data collection follow-up time: 6 weeks.	To describe the success of same-day discharge following vaginal hysterectomy and native-tissue colpopexy (pelvic organ prolapse surgery).	Adverse events and pain.
39	Burdette et al. (2025), USA	Setting: One hospital. Sample size: 1029. Data collection follow-up time: 60 days.	To evaluate safety of same-day discharge minimally invasive hysterectomy for endometrial cancer and intraepithelial neoplasia in patients with and without morbid obesity.	Complications after surgery.
40	Dieter et al. (2022), USA	Setting: The American College of Surgeons National Surgical Quality Improvement Program database. Sample size: 3717. Data collection follow-up time: 30 days.	To examine correlation between length of stay and postoperative complications following minimally invasive apical pelvic organ prolapse repair.	Postoperative complications.
41	Milman et al. (2024), Canada	Setting: The American College of Surgeons National Surgical Quality Improvement Program database. Sample size: 11,334. Data collection follow-up time: 30 days.	To assess same-day discharge and morbidity after minimally invasive hysterectomy for oncologic indication.	Postoperative morbidity.

### 3.2 Surgery characteristics

#### 3.2.1 Surgical approaches

An overview of the surgical approaches in the included studies and the treatments conducted with each approach is presented in Table 2. Four studies included surgeries by vaginal approach only (study IDs 7, 11, 37, 38). Of the 37 studies that included laparoscopies, 20 specified that robot assisted surgeries were conducted (study IDs 2, 3, 9, 10, 12, 14, 17–20, 22–24, 28, 33–35, 39–41). While three studies reported to have included laparoscopic assisted vaginal hysterectomy (study IDs 24, 35, 41), two studies specified to have excluded this surgical approach (study IDs 13, 29). Furthermore, four of the studies that included hysterectomy by laparoscopic approach noted to have performed vaginal specimen retrieval (study IDs 15, 23, 32, 33). Mini laparotomies were usually conducted in combination with laparoscopy and for specimen retrieval in studies including hysterectomy (study IDs 6, 15, 22, 23, 26), salpingo-oophorectomy (study IDs 15, 22, 23, 26), lymphadenectomy (study IDs 22, 23) and myomectomy (study ID 21).

**Table 2:** Surgical approaches and treatments in included studies.

SURGICAL APPROACH AND TREATMENT	STUDY ID	NUMBER OF STUDIES
**Laparoscopic** Hysterectomy Salpingo-oophorectomy Pelvic organ prolapse surgery Lymphadenectomy Ovarian cyst removal Myomectomy Sterilisation Trachelectomy	**1–6, 8–10, 12–36, 39–41** 2–4, 6, 8, 10, 12, 13, 15–17, 19, 20, 22–30, 32–41 1, 3, 9, 10, 15, 17, 20, 22, 23, 26, 27, 33, 34, 37 2, 12, 14, 16, 18, 19, 24, 31, 35, 38, 40 10, 17, 22, 23, 27, 30, 33, 34, 41 1, 9 9, 21 5 34	**37** 32 14 11 9 2 2 1 1
**Vaginal** Pelvic organ prolapse surgery Hysterectomy Salpingo-oophorectomy Trachelectomy	**2, 7, 11, 14, 16, 19, 24, 27, 35–38, 40** 2, 7, 11, 14, 16, 19, 24, 27, 35, 40 2, 7, 16, 19, 24, 35–38, 40 37 27	**13** 10 10 1 1

Note: Information regarding surgical approaches are highlighted in bold text.

#### 3.2.2 Surgical treatments

**Fig. 2:**
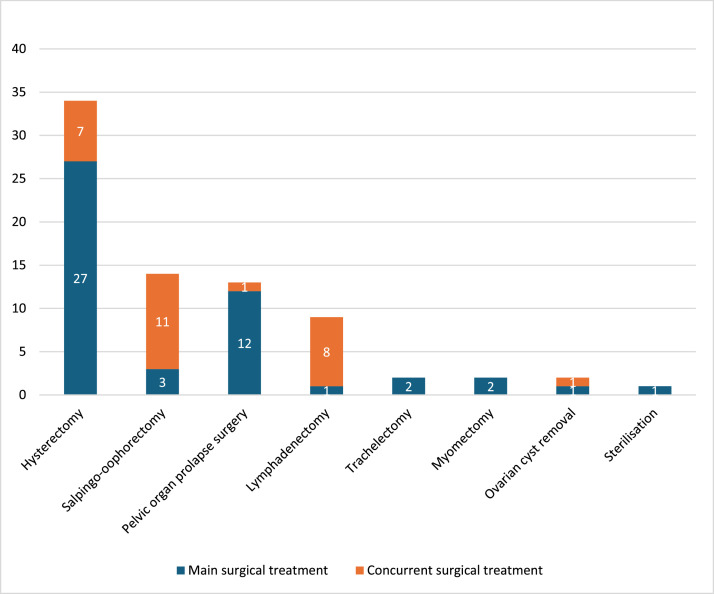
The number of studies including the surgical treatments.

The main surgical treatments conducted were hysterectomy, pelvic organ prolapse surgery, salpingo-oophorectomy, lymphadenectomy, trachelectomy, myomectomy, ovarian cyst removal and sterilisation. These treatments were also conducted concurrently with one another, as shown in Fig. 2. Other concurrent gynaecological and non-gynaecological treatments were procedures for urinary incontinence (study IDs 11, 12, 14, 16, 19, 38), hysteroscopy (study IDs 9, 21), cystoscopy (study ID 17), exploration of the abdominal cavity, salpingectomy (study IDs 9, 38), oophorectomy (study ID 38), adhesiolysis (study IDs 1, 22), ureterolysis (study ID 22), chromotubation, resection of endometriosis (study ID 9), appendectomy (study IDs 10, 23, 34), hernia repair (study ID 22), omentectomy (study IDs 10, 23, 27, 34), omental surgery (study ID 22), omental biopsy (study ID 23), peritoneal sampling (study IDs 1, 23), sentinel lymph node biopsy (study ID 30), staging of borderline ovarian tumour (study ID 1) and debulking (removal of metastases) (study ID 34). Surgical treatments for oncologic indications were included in 13 of the studies (study IDs 1, 10, 17, 22, 23, 27, 28, 30, 32–34, 39, 41).

### 3.3 Harmful incidents

All of the identified harmful incidents are listed in Table 3, mapped under the dimensions of harm based on WHO’s framework (2010); physiological harm and psychological harm. No social harmful incidents were identified from the studies included in this scoping review, and thus none are mapped. Table 4 provides an overview of the total number of incidents reported within the main categories of harmful incidents following each of the main surgical treatments.

**Table 3:** Harmful incidents mapped under dimensions of harm, number of studies reporting the harmful incident, total number of harmful incidents reported and identification numbers (study ID) for studies reporting the harmful incident.

**HARMFUL INCIDENTS**	**NUMBER OF STUDIES REPORTING THE HARMFUL INCIDENT/ TOTAL NUMBER OF INCLUDED STUDIES**	**TOTAL NUMBER OF INCIDENTS/ TOTAL NUMBER OF PARTICIPANTS IN STUDIES REPORTING THE NUMBER OF INCIDENTS**	**STUDY ID FOR STUDIES REPORTING THE HARMFUL INCIDENT**
PHYSIOLOGICAL HARM			
**Bleeding**	**23/41**	**139/11,339 (1.23%)**	**1, 3, (5,) 6, 7, 8, 10, 13, 14, 17, 20, 21, 22, 23, 24, 25, 26, (27,) 28, 29, 30, 35, 39**
Vaginal bleeding	13/41	39/7851 (0.50 %)	3, 6, 7, 8, 13, 17, 23, 24, 25, 26, (27,) 29, 30
Intraperitoneal bleeding	4/41	7/297 (2.36 %)	6, 8, 10, 14
**Postoperative infections**	**32/41**	**1038/33,454 (3.10%)**	**2, 3, 5, 6, 7, 8, 9, 10, 11, 12, 13, 16, 17, 18, 19, 20, 21, 22, 24, 25, 26, (27,) 28, 29, 30, 33, 35, 36, 38, 39, 40, 41**
Urinary tract infection	19/41	549/29,899 (1.84 %)	2, 8, 11, 12, 13, 16, (18,) 19, 22, 24, 25, (27,) 28, (35,) 36, 38, 39, 40, 41
Surgical site infection	18/41	310/30,117 (1.03 %)	5, 6, 13, 17, (18,) 21, 22, 24, 25, 26, 29, 33, 35, 36, 38, 39, 40, 41
– vaginal cuff infection	7/41	28/1923 (1.46 %)	6, 13, 22, 25, 26, 29, 38
Pelvic infection	7/41	162/21,604 (0.75 %)	3, 9, 17, 30, 36, 40, 41
**Other infections and inflammations**	**8/41**	**56/11,434 (0.49%)**	**3, 5, 13, 22, 24, (35,) 40, 41**
Sepsis	3/41	44/10,357 (0.42 %)	24, 40, 41
Pelviperitonitis	1/41	1/153 (0.65 %)	3
Reflux oesophagitis	1/41	1/22 (4.55 %)	5
Gastritis	1/41	2/128 (1.56 %)	13
Infectious enteritis	1/41	1/128 (0.78 %)	13
Appendicitis	2/41	2/903 (0.22 %)	13, 22
Diverticulitis	1/41	1/775 (0.13 %)	22
Clostridium difficile	1/41	4/775 (0.52 %)	22
Vulvar candiditis	1/41	Not reported	(35)
**Fever**	**13/41**	**27/7133 (0.38%)**	**1, 8, 10, 13, 15, 21, 24, 25, (26, 27,) 29, 30, 37**
**Harmful incidents of the surgical site**	**19/41**	**65/24,086 (0.27%)**	**5, 13, 15, 17, 21, 22, 24, 25, (26, 27,) 29, 30, 32, 33, 35, 37, 39, 40, 41**
Surgical site dehiscence	8/41	24/1921 (1.25 %)	13, 15, 17, 22, 25, 29, 32, 33
– vaginal cuff dehiscence	7/41	13/1900 (0.68 %)	13, 15, 17, 22, 25, 29, 32
Seroma	4/41	9/1021 (0.88 %)	5, 21, 22, 37
Fistula	5/41	6/898 (0.67 %)	17, (27,) 29, 30, 32
Port-site hernia	4/41	3/7310 (0.04 %)	22, 24, (29,) 39
**Vaginal discharge**	**3/41**	**3/228 (1.32%)**	**3, 30, (35)**
**Harmful incidents of the urinary system**	**15/41**	**33/22,322 (0.15%)**	**4, 5, 7, 8, 12, 13, (20,) 24, (27,) 29, 32, (35,) 38, 40, 41**
Urologic injury	3/41	3/594 (0.51 %)	8, (20,) 29
– ureteral injury	2/41	1/67 (1.49 %)	8, (20)
– bladder injury	1/41	Not reported	(20)
Ureteral stenosis	1/41	1/31 (3.23 %)	32
Urinary retention	7/41	14/6317 (0.22 %)	4, 5, 12, 24, (27,) 29, 38
Voiding dysfunction	2/41	10/804 (1.24 %)	7, 38
Dysuria	1/41	1/128 (0.78 %)	13
Acute renal failure	3/41	3/20,557 (0.01 %)	24, 40, 41
**Harmful incidents of the gastrointestinal system**	**9/41**	**32/7424 (0.43%)**	**5, 13, (20,) 22, 23, 24, 29, 35, 38**
Constipation	4/41	6/6293 (0.10 %)	13, 23, 24, 29
Bowel injury	2/41	1/775 (0.13 %)	(20,) 22
Small bowel obstruction	2/41	9/5506 (0.16 %)	(22,) 24
Bowel herniation	1/41	1/527 (0.19 %)	29
Constipation haemorrhoids	1/41	1/22 (4.55 %)	5
**Harmful incidents of the respiratory system**	**8/41**	**29/23,099 (0.13%)**	**17, 18, 22, 24, 35, 39, 40, 41**
Pneumonia	7/41	12/19,124 (0.06 %)	17, 18, 22, 24, 39, (40,) 41
Unplanned intubation and ventilator treatment	3/41	2/11,696 (0.02 %)	18, (40,) 41
Exacerbation of obstructive pulmonary disease	1/41	5/775 (0.65 %)	22
**Harmful incidents of the circulatory system**	**12/41**	**93/23,424 (0.40%)**	**13, 17, 21, 22, 24, (26,) 28, 30, 35, 39, 40, 41**
Thromboembolic disease	11/41	65/23,166 (0.28 %)	13, 17, 21, 22, 24, (26,) 28, 30, 39, 40, 41
– venous thrombo-embolism	4/41	27/12,552 (0.22 %)	21, (26,) 39, 41
– pulmonary embolism	4/41	29/17,743 (0.16 %)	13, 22, 24, 41
Myocardial infarction	4/41	10/18,164 (0.06 %)	24, 28, 39, 41
Cardiac incident	2/41	10/11,592 (0.09 %)	35, 41
Hypertensive urgency	1/41	1/775 (0.13 %)	22
Syncope	1/41	1/775 (0.13 %)	22
**Nerve injury**	**4/41**	**5/6475 (0.08%)**	**17, 22, 24, 38**
**Lymphedema**	**3/41**	**3/914 (0.33%)**	**17, 22, 33**
**Death**	**3/41**	**8/11,747 (0.07%)**	**17, 28, 41**
**Pain**	**22/41**	**192/9585 (2.00%)**	**1, 3, 5, 7, 8, 11, 12, 13, 17, 20, 22, 24, 25, (26, 27,) 29, 30, (31,) 32, 35, 37, 38**
Abdominal pain	5/41	47/1031 (4.56 %)	1, 5, 22, 30, 32
Surgical site pain	1/41	Not reported	(1)
Shoulder pain	1/41	52/138 (37.68 %)	1
Pain due to postoperative ureteral stenosis	1/41	1/21 (4.76 %)	32
Pain due to tethering of the sacral nerve	1/41	1/258 (0.39 %)	35
Chest pain	1/41	5/5506 (0.09 %)	24
**Other physiological harmful incidents**	**15/41**	**119/7975 (1.49%)**	**1, 5, 10, 12, (20,) 22, 24, 25, (26,) 27, 29, 31, 34, 35, 37**
Abdominal swelling	1/41	30/138 (21.74 %)	1
Headache	3/41	8/418 (1.91 %)	1, 5, 35
Spinal headache (presumed)	1/41	1/35 (2.86 %)	37
Backache and cramp	1/41	1/22 (4.55 %)	5
Dizziness	4/41	5/1065 (0.47 %)	5, 12, 22, 35
Nausea	7/41	13/5804 (0.22 %)	1, (20, 22,) 24, 27, (29,) 31
Vomiting	5/41	20/5803 (0.34 %)	(22,) 24, 25, (29, 37)
Decreased oral intake	1/41	Not reported	(22)
Dehydration	1/41	1/5506 (0.02 %)	24
Fatigue	1/41	31/138 (22.46 %)	1
Soft palate ecchymosis	1/41	1/775 (0.13 %)	22
Pelvic fluid collection	1/41	1/90 (1.11 %)	34
Obstructive ureteral stone	1/41	1/775 (0.13 %)	22
Cutaneous rash after antibiotics	1/41	Not reported	(26)
Medication misuse	1/41	1/157 (0.64 %)	10
Allergic reaction	1/41	2/775 (0.26 %)	22
**Physiological harmful incidents identified by patients**	**5/41**	**52/2007 (2.59%)**	**7, 19, 20, 29, 30**
Suspected infection	1/41	23/728 (3.16 %)	7
Suspected recurrence	1/41	8/728 (1.10 %)	7
Problems related to the Foley catheter	1/41	7/59 (11.86 %)	19
Problems related to the surgical site and wound care	3/41	14/1220 (1.15 %)	20, 29, 30
PSYCHOLOGICAL HARM			
**Anxiety**	**1/41**	**6/138 (4.35%)**	**1**

Notes: The main categories of harmful incidents are highlighted in bold text, under which the subcategories of harmful incidents are listed. Studies in parentheses are not included in the count of total numbers of incidents and participants, either to avoid double reporting from overlapping samples (study IDs 27 and 30) or because the number of incidents were not reported from the study.

**Table 4:** Number of harmful incidents after the various types of surgical treatment.

HARMFUL INCIDENTS - MAIN CATEGORIES	BLEEDING	POSTOPERATIVE INFECTIONS	OTHER INFECTIONS/ INFLAMMATIONS	FEVER	SURGICAL SITE PROBLEMS	VAGINAL DISCHARGE	URINARY SYSTEM	GASTROINTESTINAL	RESPIRATORY	CIRCULATORY SYSTEM	NERVE INJURY	LYMPHEDEMA	DEATH	PAIN	OTHER PHYSIOLOGICAL HARM	PHYSIOLOGICAL HARMFUL INCIDENTS IDENTIFIED BY PATIENTS	ANXIETY	STUDY ID OF STUDIES THAT INCLUDED THE SURGICAL TREATMENT
Hysterectomy	87	1034	55	19	60	3	32	31	28	92	5	3	8	98	40	52		2, 3, 4, 6, 7, 8, 10, 11, 12, 13, 15, 16, 17, 19, 20, 22, 23, 24, 25, 26, 27, 28, 29, 30, 32, 33, 34, 35, 36, 37, 38, 39, 40, 41
Pelvic organ prolapse surgery	32	257	15	4	13	X	17	23	13	18	2			60	14	38		2, 7, 11, 12, 14, 16, 18, 19, 24, 31, 35, 38, 40
Salpingo- oophorectomy	79	63	7	13	29	1	X	4	10	4	3	3	1	106	80		6	1, 3, 9, 10, 15, 17, 22, 23, 26, 27, 33, 34, 37
Lymphadenectomy	19	482	35	3	34	2	1	4	15	64	2	3	7	6	8	2		10, 17, 22, 23, 27, 30, 33, 34, 41
Trachelectomy	7	X		7	6		X							2	2			27, 34
Myomectomy	1	4		5	4					1								9, 21
Ovarian cyst removal	50	1		3										92	71		6	1, 9
Sterilisation	X	19	1		1		10	2			1			8	3			5

Note: Presented numbers are the total numbers of harmful incidents reported in studies that included the type of surgery, either as main or concurrent treatment. For studies that included multiple types of surgeries, the number of harmful incidents reported from those studies were added to the counts for all of the included surgical treatments. Studies that did not report number of incidents are also included in the last column. An X in the table indicates that the harmful incident was reported to have occurred in studies listed in the last column but the number of incidents was not reported.

Out of all the main categories of harmful incidents, postoperative infections were reported across the highest number of studies (32/41), followed by bleeding (23/41) and pain (22/41). Urinary tract infection was the subcategory of postoperative infections reported both in the highest number of studies (19/41) and with the highest number of incidents (549/1038 total incidents of postoperative infections), followed by surgical site infection (18/41 studies, 310/1038 total incidents of postoperative infections). The only specified surgical site of infection was the vaginal cuff. The vaginal cuff was also the only specified surgical site where dehiscence was reported. Another identified surgical site problem was fistula. Fistulae were reported from five studies, of which four specified that the fistulae were connected to the urinary tract (study IDs 27, 29, 30, 32). In one of these studies, ureterovaginal fistulae had been the cause of vaginal discharge (study ID 30). Vaginal discharge was identified in three of the included studies. Among the studies that reported of bleeding (23/41), 13 specified vaginal bleeding to have occurred.

Regarding harmful incidents of the urinary system, urinary retention was the subcategory reported in both the highest number of studies (7/41) and with the most incidents (14/33 total incidents of the urinary system). Constipation was the subcategory of harmful incidents related to the gastrointestinal system identified from the highest number of studies (4/41), while small bowel obstruction had the highest number of incidents (9/32 total incidents of the gastrointestinal system). For subcategories of harmful incidents in the respiratory system, pneumonia was reported across the highest number of studies (7/41) and with the most incidents (12/29 total incidents of the respiratory system). Most harmful incidents of the circulatory system were mapped under the subcategory for thromboembolic disease (65/93 total incidents of the circulatory system), which included 29 incidents of pulmonary embolism and 27 incidents of venous thromboembolism. Thromboembolic disease was also the subcategory of harm in the circulatory system that had been reported across the highest number of studies (11/41).

Pain was reported from more than half of the included studies (22/41), out of which only seven specified the site or cause of pain. The highest number of incidents of pain were in the subcategory for shoulder pain (52/192 total incidents of pain), which were all identified from one study. The site of pain specified across the highest number of studies was the abdomen (5/41), which had 47 reported incidents (out of 192 total incidents of pain). The one reported incident of pain due to tethering of a sacral nerve occurred in a study that included pelvic organ prolapse surgery and had resulted in additional surgery for removal of a uterosacral suspension suture. The four incidents of nerve injury were reported from different studies which all included hysterectomy conducted by laparoscopic approach (study IDs 17, 22, 24, 38).

Five studies reported incidents of physiological harm identified by patients that do not fit under any of the other main categories of harmful incidents. Therefore, these harmful incidents are mapped in a separate main category (Table 3). Incidents of ‘suspected recurrence’, as defined in the reporting study, are assumed to refer to the recurrence of pelvic organ prolapse, the condition for which the patients were treated (study ID 7).

The incidents of lymphedema, pelvic fluid collection and death had occurred in studies that included surgical treatment for oncologic indications. One patient had died within two weeks (study ID 17) and another within 30 days after the surgery (study ID 28). For the six remaining incidents, the time from surgery to death had not been noted (study ID 41). None of the studies reported cause of death.

The only psychological harm identified to have occurred following gynaecological ambulatory surgery was anxiety, with all six incidents having been reported from one study (study ID 1). The cause of anxiety was not described in the reporting study.

## 4 Discussion

In this scoping review, harmful incidents occurring following gynaecological ambulatory surgery were identified from 41 included studies. Most of the harmful incidents were mapped under the dimension for physiological harm. Anxiety was the only psychological harm identified. None of the included studies reported of social harm (World Health Organization, 2010).

Postoperative infections, bleeding and pain were the most reported harmful incidents, both in terms of the number of studies in which they had been identified and the number of incidents reported across all the included studies. These are harmful incidents anticipated to occur following surgical treatment within gynaecology as well as other surgical specialties, and therefore expected findings in this scoping review (Dencker et al., 2021; Kaya et al., 2021; Thurston et al., 2023). All incidents of vaginal bleeding and vaginal cuff infection were reported form studies in which hysterectomy was conducted, either by laparoscopic or vaginal approach, and all incidents of vaginal cuff dehiscence occurred in studies that included laparoscopic hysterectomy. When performing total hysterectomy, a circumferential incision is made around the cervix to remove the uterus, after which the incision at the vaginal top is sutured (Rothrock, 2023). Hence, bleeding, infection and dehiscence of the vaginal cuff can occur postoperatively. Hysterectomies were also conducted in all studies reporting of fistulae connected to the urinary tract. Previous research has found hysterectomy to be the most common gynaecological cause of vesicovaginal fistula formation (Shrestha et al., 2022).

There were no details to describe the reported incidents of nerve injury, back or shoulder pain, except that all had been identified from studies including laparoscopy. During laparoscopic gynaecological surgery, the patients are typically placed in Trendelenburg and lithotomy positioning (Rothrock, 2023). A systematic review has shown that peripheral nerve injuries of the upper extremities are most common after surgery in the Trendelenburg position, and peripheral nerve injuries of the lower extremities are most common following a lithotomy positioning (Bentsen et al., 2024). The same systematic review also found that back and shoulder pain was related to the Trendelenburg position (Bentsen et al., 2024). Shoulder pain is a common harmful incident associated with residual gas following laparoscopic surgery. Some research has also suggested that improper surgical positioning can worsen the shoulder pain (Zhao et al., 2025).

The eight incidents of death occurred after ambulatory surgery for oncologic indications, which was still concluded in the reporting studies to be as safe as in-patient treatment. Other research has found disseminated cancer to be among the most significant preoperative predictors of postoperative mortality (Alder et al., 2023). Furthermore, studies have shown that postoperative harmful incidents increased the risk of death, both after planned surgery in general and ambulatory surgery in particular (Alder et al., 2023; A. J. Fowler et al., 2022). In the current study, however, the cause of death and occurrence of other harmful incidents in these patients were not reported.

The one reported incident of medication misuse of diclofenac had resulted in readmission of the patient. Other research has shown that in outpatient settings such as ambulatory surgery practice, medication related harmful incidents constitute major safety issues and are commonly the reason for hospital readmissions (de Bienassis et al., 2022; Levine et al., 2024; Wasserman et al., 2024). Therefore, the scarcity of harmful incidents related to medications in this scoping review is interesting and indicates there is a gap of knowledge.

In the study reporting anxiety, which is the only psychological harm identified in this scoping review, part of the sample underwent salpingo-oophorectomy. Anxiety following oophorectomy is suggested to be a psychological effect of surgical menopause due to hormonal changes resulting from removal of the ovaries (Gölbaşı et al., 2023; Trister et al., 2021). No social harm was identified in this scoping review. According to WHO (2010), limitations and restrictions on participation in society due to past or present harm are considered social harm. Qualitative research has shown that patients experience dependency on others, restricted mobility and feelings of abandonment during the recovery at home following ambulatory surgery treatments within various surgical specialties (Larsson et al., 2022; Sig et al., 2025; Thoen et al., 2023). This indicates that social harm does occur in ambulatory surgery practice but is likely underreported.

### 4.1 Strengths and limitations

Inconsequent definition of length of stay in ambulatory surgery practice can affect the findings of studies on harmful incidents (Bovonratwet et al., 2017). It is therefore a strength that only studies in which patients were discharged on the same calendar day as undergoing the surgery were included in this scoping review, ensuring that the findings are relevant to the concept of interest. However, the included studies reported harmful incidents in different ways. For example, while some studies focused on specific harmful incidents, others only reported the main category of harmful incidents without further details. Also, the number of incidents were not always provided. Furthermore, inconsistent definitions and terminologies were used for the harmful incidents studied. These issues are known to challenge assessments of surgical quality and affected the analysis and findings of this scoping review (Dindo and Clavien, 2008).

Several of the studies included multiple surgical approaches and treatments, which were often conducted concurrently. Harmful incidents occurring following the various approaches and treatments were not differentiated in the study results. Therefore, there are limited findings from this scoping review to provide detailed knowledge on harmful incidents that were specific to each of the surgical treatments and approaches. Moreover, as the included studies were all conducted in western and developed countries, the findings may not reflect harmful incidents occurring in parts of the world where gynaecological ambulatory surgery is practiced differently or in countries with lower socioeconomic development. Most of the included studies used data from medical records and registries that were not particular to gynaecological or ambulatory surgery. Consequently, this scoping review most likely does not provide a complete overview of harmful incidents that are particular to gynaecological or ambulatory surgery. Furthermore, the predominance of studies based on registries and medical records and lack of qualitative studies may explain why evidence regarding the occurrence of psychological and social harm was mostly absent.

No qualitative studies were eligible in this scoping review as those identified as potentially relevant included less extensive gynaecological procedures or surgeries of other surgical specialties. This is considered a limitation because the experiences and meanings of patients, caregivers and healthcare professionals are important for identifying a wider spectrum of harmful incidents and opportunities for improving safety in gynaecological ambulatory surgery practice (Carayon et al., 2006; Levine et al., 2024; Salwei et al., 2023).

## 5 Conclusion

In this scoping review, most of the harmful incidents identified to have occurred following gynaecological ambulatory surgery were mapped as physiological harm, including postoperative infections, bleeding and pain. Anxiety was the only identified psychological harm, and no incidents of social harm was reported in the included studies.

### 5.1 Implications

More research is needed on psychological and social harm as well as medication related harmful incidents following gynaecological ambulatory surgery. Moreover, to expand current knowledge, qualitative studies should be conducted to explore harmful incidents that are identified by patients, their caregivers and healthcare professionals.

The findings of this scoping review can provide healthcare professionals with knowledge and awareness of harmful incidents occurring in gynaecological ambulatory surgery practice. Insight from the current study can also be used to enhance information provision to patients.

### 5.2 Deviations from the protocol

Initially, studies on ambulatory surgery within all surgical specialties were eligible. However, screening of titles and abstracts from the systematic literature search showed this would have resulted in an extensive volume of studies and data to be reviewed. After discussion between authors CWT, MKG, SW and SBB, the scope was thus limited to gynaecological ambulatory surgeries.

## CRediT authorship contribution statement

**Cathrine Ween Thoen:** Writing – review & editing, Writing – original draft, Visualization, Validation, Project administration, Methodology, Investigation, Formal analysis, Data curation, Conceptualization. **Malin Knutsen Glette:** Writing – review & editing, Validation, Supervision, Methodology, Investigation, Conceptualization. **Siri Wiig:** Writing – review & editing, Validation, Supervision, Conceptualization. **Kim Christian Danielsson:** Writing – review & editing, Validation. **Signe Berit Bentsen:** Writing – review & editing, Validation, Supervision, Project administration, Methodology, Investigation, Data curation, Conceptualization.

## Declaration of competing interest

The authors declare that they have no known competing financial interests or personal relationships that could have appeared to influence the work reported in this paper.

## Appendix Supplementary materials

Image, application 1

Image, application 2

Image, application 3

Image, application 4

Image, application 5
