# Supplementary material for: Harmful incidents following gynaecological ambulatory surgery: A scoping review
Source: Int J Nurs Stud Adv. 2026 Jan 7;10:100487. doi: 10.1016/j.ijnsa.2026.100487 (PMC12969120; doi:10.1016/j.ijnsa.2026.100487)
Supplement: Supplementary file 3 [file mmc3.docx]

**Supplementary material 2: The systematic literature search**

| MEDLINE | ORIGINAL SEARCH  Search date: May 26, 2023.  Records retrieved: 2097 (prior to removal of duplicates).  Comments: (None.) | UPDATED SEARCH  Search date: February 24, 2025.  Records retrieved: 397 (prior to removal of duplicates).  Comments: (None.) |
| --- | --- | --- |
| Database: | Ovid MEDLINE(R) and Epub Ahead of Print, In-Process, In-Data-Review & Other Non-Indexed Citations and Daily <1946 to May 25, 2023> | Ovid MEDLINE(R) ALL <1946 to February 20, 2025> |
| Search Strategy | | |
| 1 | exp Ambulatory Surgical Procedures/ (13112) | exp Ambulatory Surgical Procedures/ (13642) |
| 2 | Outpatient Clinics, Hospital/ (15846) | Outpatient Clinics, Hospital/ (15867) |
| 3 | exp Surgicenters/ (2001) | exp Surgicenters/ (2014) |
| 4 | (ambulatory adj (surg* or procedure*)).ti,ab. (5272) | (ambulatory adj (surg* or procedure*)).ti,ab. (5791) |
| 5 | (day adj (surg* or case*)).ti,ab. (7122) | (day adj (surg* or case*)).ti,ab. (7662) |
| 6 | (same adj day).ti,ab. (18142) | (same adj day).ti,ab. (20535) |
| 7 | (outpatient adj (procedure* or surg*)).ti,ab. (5210) | (outpatient adj (procedure* or surg*)).ti,ab. (5697) |
| 8 | (surgical adj day adj care).ti,ab. (46) | (surgical adj day adj care).ti,ab. (49) |
| 9 | or/1-8 (56429) | 1 or 2 or 3 or 4 or 5 or 6 or 7 or 8 (60259) |
| 10 | exp Patient Safety/ (25351) | exp Patient Safety/ (27125) |
| 11 | exp Patient Harm/ (219) | exp Patient Harm/ (244) |
| 12 | exp Treatment Failure/ (37378) | exp Treatment Failure/ (38293) |
| 13 | (patient adj (safety or harm)).ti,ab. (36907) | (patient adj (safety or harm)).ti,ab. (43220) |
| 14 | (harmful adj incident*).ti,ab. (36) | (harmful adj incident*).ti,ab. (47) |
| 15 | (adverse adj (event* or outcome*)).ti,ab. (255361) | (adverse adj (event* or outcome*)).ti,ab. (302170) |
| 16 | (safety adj (outcome* or event*)).ti,ab. (8619) | (safety adj (outcome* or event*)).ti,ab. (11314) |
| 17 | (surgical adj safety).ti,ab. (1427) | (surgical adj safety).ti,ab. (1778) |
| 18 | (treatment adj failure*).ti,ab. (31789) | (treatment adj failure*).ti,ab. (35417) |
| 19 | (unexpected adj issue*).ti,ab. (54) | (unexpected adj issue*).ti,ab. (59) |
| 20 | (unsuccessful adj surg*).ti,ab. (578) | (unsuccessful adj surg*).ti,ab. (618) |
| 21 | or/10-20 (369655) | 10 or 11 or 12 or 13 or 14 or 15 or 16 or 17 or 18 or 19 or 20 (428329) |
| 22 | 9 and 21 (2097) | 9 and 21 (2404) |
| 23 |  | limit 22 to yr="2023 -Current" (397) |

| Embase | ORIGINAL SEARCH  Search date: May 26, 2023.  Records retrieved: 4759 (prior to removal of duplicates).  Comments: (None.) | UPDATED SEARCH  Search date: February 24, 2025.  Records retrieved: 903 (prior to removal of duplicates).  Comments: (None.) |
| --- | --- | --- |
| Database: | Embase <1974 to 2023 Week 20> | Embase <1974 to 2025 Week 07> |
| Search Strategy | | |
| 1 | ambulatory surgery/ (18914) | ambulatory surgery/ (20034) |
| 2 | (ambulatory adj (surg* or procedure*)).ti,ab. (7524) | (ambulatory adj (surg* or procedure*)).ti,ab. (8109) |
| 3 | (day adj (surg* or case*)).ti,ab. (12210) | (day adj (surg* or case*)).ti,ab. (13071) |
| 4 | (same adj day).ti,ab. (32923) | (same adj day).ti,ab. (36819) |
| 5 | (outpatient adj (procedure* or surg*)).ti,ab. (7658) | (outpatient adj (procedure* or surg*)).ti,ab. (8303) |
| 6 | (surgical adj day adj care).ti,ab. (65) | (surgical adj day adj care).ti,ab. (65) |
| 7 | surgicent*.ti,ab. (118) | surgicent*.ti,ab. (120) |
| 8 | or/1-7 (64745) | 1 or 2 or 3 or 4 or 5 or 6 or 7 (70693) |
| 9 | exp patient safety/ (162310) | exp patient safety/ (183451) |
| 10 | exp patient harm/ (2813) | exp patient harm/ (3415) |
| 11 | exp treatment failure/ (175967) | exp treatment failure/ (217691) |
| 12 | (patient adj (safety or harm)).ti,ab. (55636) | (patient adj (safety or harm)).ti,ab. (62930) |
| 13 | (harmful adj incident*).ti,ab. (58) | (harmful adj incident*).ti,ab. (66) |
| 14 | (adverse adj (event* or outcome*)).ti,ab. (458045) | (adverse adj (event* or outcome*)).ti,ab. (521695) |
| 15 | (safety adj (outcome* or event*)).ti,ab. (15820) | (safety adj (outcome* or event*)).ti,ab. (19398) |
| 16 | (surgical adj safety).ti,ab. (2053) | (surgical adj safety).ti,ab. (2444) |
| 17 | (treatment adj failure*).ti,ab. (51282) | (treatment adj failure*).ti,ab. (55703) |
| 18 | (unexpected adj issue*).ti,ab. (72) | (unexpected adj issue*).ti,ab. (76) |
| 19 | (unsuccessful adj surg*).ti,ab. (735) | (unsuccessful adj surg*).ti,ab. (780) |
| 20 | or/9-19 (817914) | 9 or 10 or 11 or 12 or 13 or 14 or 15 or 16 or 17 or 18 or 19 (941152) |
| 21 | 8 and 20 (4759) | 8 and 20 (5428) |
| 22 |  | limit 21 to yr="2023 -Current" (903) |

| CINAHL | ORIGINAL SEARCH  Search date: May 26, 2023.  Records retrieved: 1492 (prior to removal of duplicates).  Comments: (None.) | UPDATED SEARCH  Search date: February 24, 2025.  Records retrieved: 185 (prior to removal of duplicates).  Comments: Search number 21 was limited to publications from 2023. |
| --- | --- | --- |
| Database: | Interface - EBSCOhost Research Databases  Search Screen - Advanced Search  Database – CINAHL  Expanders - Apply equivalent subjects  Search modes - Boolean/Phrase | (No details.) |
| Search Strategy | | |
| 1 | (MH "Ambulatory Surgery") (6852) | (MH "Ambulatory Surgery") (6965) |
| 2 | (MH "Surgicenters") (3533) | (MH "Surgicenters") (3603) |
| 3 | TI ( ambulatory W0 (surg* or procedure*) ) OR AB ( ambulatory W0 (surg* or procedure*) ) (2427) | TI ( ambulatory W0 (surg* or procedure*) ) OR AB (ambulatory W0 (surg* or procedure*) ) (2303) |
| 4 | TI ( day W0 (surg* or case*) ) OR AB ( day W0 (surg* or case*) ) (3118) | TI ( day W0 (surg* or case*) ) OR AB ( day W0 (surg* or case*) ) (3141) |
| 5 | TI same W0 day OR AB same W0 day (5830) | TI same W0 day OR AB same W0 day (6170) |
| 6 | TI ( outpatient W0 (procedure* or surg*) ) OR AB ( outpatient W0 (procedure* or surg*) ) (1624) | TI ( outpatient W0 (procedure* or surg*) ) OR AB (outpatient W0 (procedure* or surg*) ) (1657) |
| 7 | TI surgical W0 day W0 care OR AB surgical W0 day W0 care (11) | TI surgical W0 day W0 care OR AB surgical W0 day W0 care (11) |
| 8 | S1 OR S2 OR S3 OR S4 OR S5 OR S6 OR S7 (17962) | S1 OR S2 OR S3 OR S4 OR S5 OR S6 OR S7 (18520) |
| 9 | (MH "Patient Safety+") (143041) | (MH "Patient Safety+") (161728) |
| 10 | (MH "Treatment Failure+") (16030) | (MH "Treatment Failure+") (16508) |
| 11 | TI ( patient W0 (safety or harm) ) OR AB ( patient W0 (safety or harm) ) (28493) | TI ( patient W0 (safety or harm) ) OR AB ( patient W0 (safety or harm) ) (29375) |
| 12 | TI harmful W0 incident* OR AB harmful W0 incident* (32) | TI harmful W0 incident* OR AB harmful W0 incident* (35) |
| 13 | TI ( adverse W0 (event* or outcome*) ) OR AB ( adverse W0 (event* or outcome*) ) (85427) | TI ( adverse W0 (event* or outcome*) ) OR AB ( adverse W0 (event* or outcome*) ) (90168) |
| 14 | TI ( safety W0 (outcome* or event*) ) OR AB ( safety W0 (outcome* or event*) ) (3528) | TI ( safety W0 (outcome* or event*) ) OR AB ( safety W0 (outcome* or event*) ) (3970) |
| 15 | TI surgical W0 safety OR AB surgical W0 safety (598) | TI surgical W0 safety OR AB surgical W0 safety (611) |
| 16 | TI treatment W0 failure* OR AB treatment W0 failure* (6894) | TI treatment W0 failure* OR AB treatment W0 failure* (7230) |
| 17 | TI unexpected W0 issue* OR AB unexpected W0 issue* (23) | TI unexpected W0 issue* OR AB unexpected W0 issue* (27) |
| 18 | TI unsuccessful W0 surg* OR AB unsuccessful W0 surg* (104) | TI unsuccessful W0 surg* OR AB unsuccessful W0 surg* (104) |
| 19 | S9 OR S10 OR S11 OR S12 OR S13 OR S14 OR S15 OR S16 OR S17 OR S18 (247600) | S9 OR S10 OR S11 OR S12 OR S13 OR S14 OR S15 OR S16 OR S17 OR S18 (268935) |
| 20 | S8 AND S19 (1492) | S8 AND S19 (1611) |
| 21 |  | S8 AND S19 (185) |

| Cochrane Central | ORIGINAL SEARCH  Search date: May 26, 2023.  Records retrieved: 25 reviews, 901 trials (prior to removal of duplicates).  Comments: Only the reviews were imported. | UPDATED SEARCH  Search date: February 24, 2025.  Records retrieved: 2 reviews, 144 trials (prior to removal of duplicates).  Comments: (None.) |
| --- | --- | --- |
| Database: | (No details.) | (No details.) |
| Search Strategy | | |
| 1 | MeSH descriptor: [Ambulatory Surgical Procedures] explode all trees (1659) | MeSH descriptor: [Ambulatory Surgical Procedures] explode all trees (1760) |
| 2 | MeSH descriptor: [Outpatient Clinics, Hospital] explode all trees (706) | MeSH descriptor: [Outpatient Clinics, Hospital] explode all trees (733) |
| 3 | MeSH descriptor: [Surgicenters] explode all trees (7) | MeSH descriptor: [Surgicenters] explode all trees (8) |
| 4 | (ambulatory NEXT (surg* or procedure*)):ti,ab,kw (Word variations have been searched) (3069) | (ambulatory NEXT (surg* or procedure*)):ti,ab,kw (Word variations have been searched) (3239) |
| 5 | (day NEXT (surg* orprocedure*)):ti,ab,kw (Word variations have been searched) (2051) | (day NEXT (surg* or case*)):ti,ab,kw (Word variations have been searched) (2223) |
| 6 | (same NEXT day):ti,ab,kw (Word variations have been searched) (3506) | (same NEXT day):ti,ab,kw (Word variations have been searched) (3979) |
| 7 | (outpatient NEXT (procedure* or surg*));ti,ab,kw (Word variations have been searched) (1036) | (outpatient NEXT (procedure* or surg*)):ti,ab,kw (Word variations have been searched) (1127) |
| 8 | (surgical NEXT day NEXT care):ti,ab,kw (Word variations have been searched) (16) | (surgical NEXT day NEXT care):ti,ab,kw (Word variations have been searched) (16) |
| 9 | #1 OR #2 OR #3 OR #4 OR #5 OR #6 OR #7 OR #8 (9094) | #1 OR #2 OR #3 OR #4 OR #5 OR #6 OR #7 OR #8 (9936) |
| 10 | MeSH descriptor: [Patient Safety] explode all trees (1529) | MeSH descriptor: [Patient Safety] explode all trees (1036) |
| 11 | MeSH descriptor: [Patients Harm] explode all trees (15) | MeSH descriptor: [Patient Harm] explode all trees (6) |
| 12 | MeSH descriptor: [Treatment Failure] explode all trees (5142) | MeSH descriptor: [Treatment Failure] explode all trees (4052) |
| 13 | (patient NEXT (safety or harm)):ti,ab,kw (Word variations have been searched) (8796) | (patient NEXT (safety or harm)):ti,ab,kw (Word variations have been searched) (9623) |
| 14 | (harmful NEXT incident*):ti,ab,kw (Word variations have been searched) (2) | (harmful NEXT incident*):ti,ab,kw (Word variations have been searched) (2) |
| 15 | (adverse NEXT (event* or outcome*)):ti,ab,kw (Word variations have been searched) (143685) | (adverse NEXT (event* or outcome*)):ti,ab,kw (Word variations have been searched) (164807) |
| 16 | (safety NEXT (outcome* or event*)):ti,ab,kw (Word variations have been searched) (5936) | (safety NEXT (outcome* or event*)):ti,ab,kw (Word variations have been searched) (6296) |
| 17 | (surgical NEXT safety):ti,ab,kw (Word variations have been searched) (152) | (surgical NEXT safety):ti,ab,kw (Word variations have been searched) (184) |
| 18 | (treatment NEXT failure*):ti,ab,kw (Word variations have been searched) (19171) | (treatment NEXT failure*):ti,ab,kw (Word variations have been searched) (19952) |
| 19 | (unexpected NEXT issue*):ti,ab,kw (Word variations have been searched) (5) | (unexpected NEXT issue*):ti,ab,kw (Word variations have been searched) (7) |
| 20 | (unsuccessful NEXT surg*):ti,ab,kw (Word variations have been searched) (21) | (unsuccessful NEXT surg*):ti,ab,kw (Word variations have been searched) (21) |
| 21 | #10 OR #11 OR #12 OR #13 OR #14 OR #15 OR #16 OR #17 OR #18 OR #19 OR #20 (168401) | #10 OR #11 OR #12 OR #13 OR #14 OR #15 OR #16 OR #17 OR #18 OR #19 OR #20 (191464) |
| 22 | #9 and #21 (926) | #9 AND #21 (1044) |

| Scopus | ORIGINAL SEARCH  Search date: May 31, 2023.  Records retrieved: 4593 (prior to removal of duplicates).  Comments: (None.) | UPDATED SEARCH  Search date: February 24, 2025.  Records retrieved: 912 (prior to removal of duplicates).  Comments: (None.) |
| --- | --- | --- |
| Database: | (No details.) | (No details.) |
| Search Strategy | | |
| 1 | TITLE-ABS-KEY ( ambulatory W/0 ( surg* OR procedure* ) ) (20083) | TITLE-ABS-KEY ( ambulatory W/0 ( surg* OR procedure* ) ) (21514) |
| 2 | TITLE-ABS-KEY ( day W/0 ( surg* OR case* ) ) (11047) | TITLE-ABS-KEY ( day W/0 ( surg* OR case* ) ) (11993) |
| 3 | TITLE-ABS-KEY ( same W/0 day ) (24809) | TITLE-ABS-KEY ( same W/0 day ) (28010) |
| 4 | TITLE-ABS-KEY ( outpatient W/0 (procedure* OR surg* ) ) (7559) | TITLE-ABS-KEY ( outpatient W/0 (procedure* OR surg* ) ) (8402) |
| 5 | TITLE-ABS-KEY ( surgical W/0 day W/0 care ) (76) | TITLE-ABS-KEY ( surgical W/0 day W/0 care ) (81) |
| 6 | ( TITLE-ABS-KEY ( ambulatory W/0 ( surg*OR procedure* ) ) ) OR (TITLE-ABS-KEY ( day W/0 ( surg*OR case* ) ) ) OR (TITLE-ABS-KEY ( same W/0 day ) ) OR (TITLE-ABS-KEY ( outpatient W/0 (procedure* OR surg* ) ) ) OR (TITLE-ABS-KEY ( surgical W/0 day W/0 care ) ) (55198) | ( TITLE-ABS-KEY ( ambulatory W/0 ( surg*OR procedure* ) ) ) OR (TITLE-ABS-KEY ( day W/0 ( surg*OR case* ) ) ) OR (TITLE-ABS-KEY ( same W/0 day ) ) OR (TITLE-ABS-KEY ( outpatient W/0 (procedure* OR surg* ) ) ) OR (TITLE-ABS-KEY ( surgical W/0 day W/0 care ) ) (60804) |
| 7 | TITLE-ABS-KEY ( patient W/0 ( safety OR harm ) ) (172198) | TITLE-ABS-KEY ( patient W/0 ( safety OR harm ) ) (195749) |
| 8 | TITLE-ABS-KEY ( harmful W/0 incident* ) (87) | TITLE-ABS-KEY ( harmful W/0 incident* ) (108) |
| 9 | TITLE-ABS-KEY ( adverse W/0 ( event* OR outcome* ) ) (494110) | TITLE-ABS-KEY ( adverse W/0 ( event* OR outcome* ) ) (584442) |
| 10 | TITLE-ABS-KEY ( safety W/0 ( outcome* OR event* ) ) (10659) | TITLE-ABS-KEY ( safety W/0 ( outcome* OR event* ) ) (14023) |
| 11 | TITLE-ABS-KEY ( surgical W/0 safety ) (1900) | TITLE-ABS-KEY ( surgical W/0 safety ) (2410) |
| 12 | TITLE-ABS-KEY ( treatment W/0 failure* ) (160566) | TITLE-ABS-KEY ( treatment W/0 failure* ) (171317) |
| 13 | TITLE-ABS-KEY ( unexpected W/0 issue* ) (202) | TITLE-ABS-KEY ( unexpected W/0 issue* ) (258) |
| 14 | TITLE-ABS-KEY ( unsuccessful W/0 surg* ) (664) | TITLE-ABS-KEY ( unsuccessful W/0 surg* ) (713) |
| 15 | ( TITLE-ABS-KEY ( patient W/0 ( safety OR harm ) ) OR ( TITLE-ABS-KEY ( harmful W/0 incident* ) ) OR ( TITLE-ABS-KEY ( adverse W/0 ( event* OR outcome* ) ) ) OR ( TITLE-ABS-KEY ( safety W/0 ( outcome* OR event* ) ) ) OR ( TITLE-ABS-KEY ( surgical W/0 safety ) ) OR ( TITLE-ABS-KEY ( treatment W/0 failure* ) ) OR ( TITLE-ABS-KEY ( unexpected W/0 issue* ) ) OR ( TITLE-ABS-KEY ( unsuccessful W/0 surg* ) ) (798407) | ( TITLE-ABS-KEY ( patient W/0 ( safety OR harm ) ) ) OR ( TITLE-ABS-KEY ( harmful W/0 incident* ) ) OR ( TITLE-ABS-KEY ( adverse W/0 ( event* OR outcome* ) ) ) OR ( TITLE-ABS-KEY ( safety W/0 ( outcome* OR event* ) ) ) OR ( TITLE-ABS-KEY ( surgical W/0 safety ) ) OR ( TITLE-ABS-KEY ( treatment W/0 failure* ) ) OR ( TITLE-ABS-KEY ( unsuccessful W/0 surg* ) ) OR ( TITLE-ABS-KEY ( unexpected W/0 issue* ) ) (920162) |
| 16 | ( ( TITLE-ABS-KEY ( ambulatory W/0 ( surg*OR procedure* ) ) ) OR (TITLE-ABS-KEY ( day W/0 ( surg*OR case* ) ) ) OR (TITLE-ABS-KEY ( same W/0 day ) ) OR (TITLE-ABS-KEY ( outpatient W/0 (procedure* OR surg* ) ) ) OR (TITLE-ABS-KEY ( surgical W/0 day W/0 care ) ) ) AND ( ( TITLE-ABS-KEY ( patient W/0 ( safety OR harm ) ) OR ( TITLE-ABS-KEY ( harmful W/0 incident* ) ) OR ( TITLE-ABS-KEY ( adverse W/0 ( event* OR outcome* ) ) ) OR ( TITLE-ABS-KEY ( safety W/0 ( outcome* OR event* ) ) ) OR ( TITLE-ABS-KEY ( surgical W/0 safety ) ) OR ( TITLE-ABS-KEY ( treatment W/0 failure* ) ) OR ( TITLE-ABS-KEY ( unexpected W/0 issue* ) ) OR ( TITLE-ABS-KEY ( unsuccessful W/0 surg* ) ) ) (4593) | ( ( TITLE-ABS-KEY ( patient W/0 ( safety OR harm ) ) ) OR ( TITLE-ABS-KEY (harmful W/0 incident* ) ) OR ( TITLE-ABS-KEY ( adverse W/0 ( event* OR outcome* ) ) ) OR (TITLE-ABS-KEY ( safety W/0 (outcome* OR event* ) ) ) OR ( TITLE-ABS-KEY ( surgical W/0 safety ) ) OR ( TITLE-ABS-KEY ( treatment W/0 failure* ) ) OR (TITLE-ABS-KEY ( unsuccessful W/0 surg* ) ) OR ( TITLE-ABS-KEY (unexpected W/0 issue* ) ) ) AND ( ( TITLE-ABS-KEY ( ambulatory W/0 ( surg* OR procedure* ) ) ) OR ( TITLE-ABS-KEY ( day W/0 ( surg* OR case* ) ) ) OR ( TITLE-ABS-KEY ( same W/0 day ) ) OR ( TITLE-ABS-KEY (outpatient W/0 ( procedure* OR surg* ) ) ) OR ( TITLE-ABS-KEY ( surgical W/0 day W/0 care ) ) ) (5305) |
| 17 |  | ( ( TITLE-ABS-KEY ( patient W/0 ( safety OR harm ) ) ) OR ( TITLE-ABS-KEY (harmful W/0 incident* ) ) OR ( TITLE-ABS-KEY ( adverse W/0 ( event* OR outcome* ) ) ) OR (TITLE-ABS-KEY ( safety W/0 (outcome* OR event* ) ) ) OR ( TITLE-ABS-KEY ( surgical W/0 safety ) ) OR ( TITLE-ABS-KEY ( treatment W/0 failure* ) ) OR (TITLE-ABS-KEY ( unsuccessful W/0 surg* ) ) OR ( TITLE-ABS-KEY (unexpected W/0 issue* ) ) ) AND ( ( TITLE-ABS-KEY ( ambulatory W/0 ( surg* OR procedure* ) ) ) OR ( TITLE-ABS-KEY ( day W/0 ( surg* OR case* ) ) ) OR ( TITLE-ABS-KEY ( same W/0 day ) ) OR ( TITLE-ABS-KEY (outpatient W/0 ( procedure* OR surg* ) ) ) OR ( TITLE-ABS-KEY ( surgical W/0 day W/0 care ) ) ) AND PUBYEAR > 2022 AND PUBYEAR < 2026 (912) |

| Web of Science | ORIGINAL SEARCH  Search date: May 31, 2023.  Records retrieved: 1592 (prior to removal of duplicates).  Comments: (None.) | UPDATED SEARCH  Search date: February 24, 2025.  Records retrieved: 231 (prior to removal of duplicates).  Comments: (None.) |
| --- | --- | --- |
| Database: | (No details.) | (No details.) |
| Search Strategy | | |
| 1 | ambulatory NEAR/0 (surg* or procedure*) (Topic) (5017) | ambulatory NEAR/0 (surg* or procedure*) (Topic) (5654) |
| 2 | day NEAR/0 (surg* or case*) (Topic) (8314) | day NEAR/0 (surg* or case*) (Topic) (9254) |
| 3 | outpatient NEAR/0 (procedure* or surg*) (Topic) (5204) | outpatient NEAR/0 (procedure* or surg*) (Topic) (5965) |
| 4 | surgical NEAR/0 day NEAR/0 care (Topic) (41) | surgical NEAR/0 day NEAR/0 care (Topic) (46) |
| 5 | same* NEAR/0 day (Topic) (18922) | #5 OR #4 OR #3 OR #2 OR #1 (19195) |
| 6 | #5 OR #4 OR #3 OR #2 OR #1 (34898) | patient NEAR/0 (safety or harm) (Topic) (50184) |
| 7 | patient NEAR/0 (safety or harm) (Topic) (42446) | harmful NEAR/0 incident* (Topic) (54) |
| 8 | harmful NEAR/0 incident* (Topic) (43) | adverse NEAR/0 (event* or outcome*) (Topic) (304276) |
| 9 | adverse NEAR/0 (event* or outcome*) (Topic) (254646) | safety NEAR/0 (outcome* or event*) (Topic) (12795) |
| 10 | safety NEAR/0 (outcome* or event*) (Topic) (9623) | surgical NEAR/0 safety (Topic) (2154) |
| 11 | surgical NEAR/0 safety (Topic) (1757) | treatment NEAR/0 failure* (Topic) (37887) |
| 12 | treatment NEAR/0 failure* (Topic) (33014) | unexpected NEAR/0 issue* (Topic) (119) |
| 13 | unexpected NEAR/0 issue* (Topic) (94) | unsuccessful NEAR/0 surg* (Topic) (494) |
| 14 | unsuccessful NEAR/0 surg* (Topic) (421) | #13 OR #12 OR #11 OR #10 OR #9 OR #8 OR #6 (392978) |
| 15 | #7 OR #8 OR #9 OR #10 OR #11 OR #12 OR #13 OR #14 (329586) | #5 AND #14 (1234) |
| 16 | #6 OR #15 (1592) | #5 AND #14 and 2023 or 2024 or 2025 (Publication Years) (231) |
