# Supplementary material for: Harmful incidents following gynaecological ambulatory surgery: A scoping review
Source: Int J Nurs Stud Adv. 2026 Jan 7;10:100487. doi: 10.1016/j.ijnsa.2026.100487 (PMC12969120; doi:10.1016/j.ijnsa.2026.100487)
Supplement: Supplementary file 4 [file mmc4.docx]

**Supplementary material 3: Records excluded in screening of full text and reasons for exclusion**

| RECORDS FROM ORIGINAL AND UPDATED SYSTEMATIC LITERATURE SEARCH | | | |
| --- | --- | --- | --- |
| DistillerSRreference ID | **Bibliographic information** | **Reason for exclusion** | **Number of records** |
| 28 | Abbasher, M., Ramadan, W. A., Halder, N., Mabrouk, Q., & Abdelrahman, I. (2019). Laparoscopic hysterectomy as a day case in a district general hospital (Abstract) | Wrong source | 1 |
| 105 | Ahmad, J., Ho, O. A., Carman, W. W., Thoma, A., Lalonde, D. H., & Lista, F. (2014). Assessing patient safety in Canadian ambulatory surgery facilities: A national survey. Plastic Surgery (Oakville (Ont.)), 22(1), 34–38. <https://doi.org/10.1177/229255031402200101> | Harmful incidents not reported | 2 |
| 149 | Al-Singary, W., Shergill, I. S., Allen, S. E., John, J. A., Arya, M., & Patel, H. R. H. (2007). Trans-obturator tape for incontinence: a 3-year follow-up. Urologia Internationalis, 78(3), 198-201. | Wrong surgeries | 3 |
| 304 | Antoun, L., Smith, P., Gupta, J. K., & Clark, T. J. (2017). The feasibility, safety, and effectiveness of hysteroscopic sterilization compared with laparoscopic sterilization. *American Journal of Obstetrics and Gynecology*, *217*(5), 570.e1-570.e6. <https://doi.org/10.1016/j.ajog.2017.07.011> | Harmful incidents not reported | 4 |
| 314 | Aranaz, J. M., Ivorra, F., Compañ, A. F., Miralles, J. J., Gea, M. T., Limón, R., Requena, J., Rey, M., & García, R. (2008). Efectos adversos en cirugía mayor ambulatoria [Adverse events in ambulatory surgical procedures]. *Cirugia espanola*, *84*(5), 273–278. <https://doi.org/10.1016/s0009-739x(08)75920-7> | Wrong population | 5 |
| 383 | Aunier, J., Reboul, Q., & Chauleur, C. (2022). Evaluation of surgical treatment of genital prolapse using synthetic mesh in an outpatient procedure and patient satisfaction. *Journal of Gynecology Obstetrics and Human Reproduction*, *51*(3), 102312–102312. <https://doi.org/10.1016/j.jogoh.2022.102312> | Unable to extract data | 6 |
| 514 | Barros, A. L., Duque, M. R., Nunes, F., Ribeiro, S., Ventura, C., & Assunção, J. P. (2014). Five year retrospective study of unanticipated admission after ambulatory surgery: 2AP2-2. *European Journal of Anaesthesiology\| EJA*, *31*, 27. (Abstract) | Wrong source | 7 |
| 594 | Berger G. S. (1994). Outpatient pelvic laparotomy. *The Journal of reproductive medicine*, *39*(8), 569–574. (Abstract) | Wrong source | 8 |
| 675 | Blackwell, L. A. (2014). A Successful Life Safety Survey in an Ambulatory Surgery Center. *AORN Journal*, *99*(3), 431–434. <https://doi.org/10.1016/j.aorn.2014.01.001> | Wrong population | 9 |
| 727 | Bongiovanni, T., Parzynski, C., Ranasinghe, I., Steinman, M. A., & Ross, J. S. (2021). Unplanned hospital visits after ambulatory surgical care. *PloS One*, *16*(7), e0254039–e0254039. <https://doi.org/10.1371/journal.pone.0254039> | Unable to extract data | 10 |
| 802 | Bretschneider, C. E., Luchristt, D., Kenton, K., & Sheyn, D. (2022). Evaluating the association of same-day discharge following minimally invasive surgery for prolapse on 30-day postoperative adverse events. *International Urogynecology Journal*, *33*(7), 1849–1856. <https://doi.org/10.1007/s00192-021-05035-y> | Wrong population | 11 |
| 836 | Bruneau, L., Randet, M., Evrard, S., Damon, A., & Laurent, F. X. (2015). Prise en charge ambulatoire de l'hystérectomie laparoscopique : évaluation de la faisabilité et de la satisfaction des patientes [Total laparoscopic hysterectomy and same-day discharge: Satisfaction evaluation and feasibility study]. *Journal de gynecologie, obstetrique et biologie de la reproduction*, *44*(9), 870–876. <https://doi.org/10.1016/j.jgyn.2015.02.003> | Harmful incidents not reported | 12 |
| 902 | Byford, S., Madhok, D., Baldan, P., Amey, G., Barber, J., Harris, R., & Walker, G. (2022). Introduction of the day case total laparoscopic hysterectomy (TLH) protocol. *Australian & New Zealand Journal of Obstetrics & Gynaecology*, *62*(6), 881–886. <https://doi.org/10.1111/ajo.13598> | Unable to extract data | 13 |
| 974 | Cappuccio, S., Li, Y., Song, C., Liu, E., Glaser, G., Casarin, J., Grassi, T., Butler, K., Magtibay, P., Magrina, J. F., Scambia, G., Mariani, A., & Langstraat, C. (2021). The shift from inpatient to outpatient hysterectomy for endometrial cancer in the United States: trends, enabling factors, cost, and safety. *International journal of gynecological cancer : official journal of the International Gynecological Cancer Society*, *31*(5), 686–693. <https://doi.org/10.1136/ijgc-2020-002192> | Unable to extract data | 14 |
| 981 | Carayon, P., Schoofs Hundt, A., Alvarado, C. J., Springman, S. R., & Ayoub, P. (2006). Patient safety in outpatient surgery: The viewpoint of the healthcare providers: Patient safety. *Ergonomics*, *49*(5–6), 470–485. | Not ambulatory surgery | 15 |
| 1015 | Cassis, C., Mukhopadhyay, S., Sule, M. M., & Kuruba, N. (2018). Feasibility of early discharge following vaginal hysterectomy with a bipolar electrocoagulation device. *International Journal of Gynecology and Obstetrics*, *142*(2), 182–186. <https://doi.org/10.1002/ijgo.12515> | Unable to extract data | 16 |
| 1019 | Castillón Vela, I. T., Redondo González, E., Linares Quevedo, A. I., Vallejo Herrador, J., Ríos González, E., Sáenz Medina, J., & Páez Borda, A. (2007). Cirugia de la incontinencia urinaria femenina en regimen de cirugia mayor ambulatoria: estudio de impacto presupuestario [Outpatient female stress urinary incontinence surgery: budget impact study]. *Archivos espanoles de urologia*, *60*(3), 267–272. <https://doi.org/10.4321/s0004-06142007000300006> | Wrong surgeries | 17 |
| 1155 | Chien, C., & Cheng, G. (2022). A community hospital’s approach towards same-day discharge after minimally invasive hysterectomies during the COVID-19 pandemic. (Abstract) | Wrong source | 18 |
| 1203 | Christiansen, U. J., Kruse, A. R., Olesen, P. G., Lauszus, F. F., Kesmodel, U. S., & Forman, A. (2019). Outpatient vs inpatient total laparoscopic hysterectomy: A randomized controlled trial. *Acta Obstetricia et Gynecologica Scandinavica*, *98*(11), 1420–1428. <https://doi.org/10.1111/aogs.13670> | Unable to extract data | 19 |
| 1212 | Chudnoff, S. G., Berman, J. M., Levine, D. J., Harris, M., Guido, R. S., & Banks, E. (2013). Outpatient procedure for the treatment and relief of symptomatic uterine myomas. *Obstetrics and Gynecology (New York. 1953)*, *121*(5), 1075–1082. <https://doi.org/10.1097/AOG.0b013e31828b7962> | Unable to extract data | 20 |
| 1300 | Collins, L. M., & Vaghadia, H. (2001). Regional anesthesia for laparoscopy. *Anesthesiology clinics of north America*, *19*(1), 43-55. (Not research) | Wrong source | 21 |
| 1345 | Cope, Z., Platte, R. O., Shah, N. M., & Leary, E. (2018). Does being discharged on the same day of urogynecologic surgery opposed to remaining inpatient overnight increase the frequency of voiding dysfunction encountered in the first 6 postoperative weeks. (Abstract) | Wrong source | 22 |
| 1375 | Courtieu, C., Cornille, A., Vaast, M., Lacombe, S., & Panel, L. (2019). Prospective feasibility study of ambulatory surgery for pelvic organ prolapse. *European Journal of Obstetrics & Gynecology and Reproductive Biology*, *236*, 36–40. <https://doi.org/10.1016/j.ejogrb.2019.01.019> | Unable to extract data | 23 |
| 1376 | Courtieu, C., Panel, L., Cornille, A. Outpatient surgery (OPS) for pelvic organ prolapse (POP): Preliminary results of pcap study. (Conference oral presentation?) | Wrong source | 24 |
| 1475 | Danilyants, N., Mamik, M. M., MacKoul, P., Does, L. Q., & Haworth, L. (2020). Laparoscopic‐assisted myomectomy: Surgery center versus outpatient hospital. *The Journal of Obstetrics and Gynaecology Research*, *46*(3), 490–498. <https://doi.org/10.1111/jog.14197> | Unable to extract data | 25 |
| 1561 | DeFrancesco M. S. (2019). Patient Safety in Outpatient Procedures. *Obstetrics and gynecology clinics of North America*, *46*(2), 379–387. <https://doi.org/10.1016/j.ogc.2019.01.012> (Not research) | Wrong source | 26 |
| 1575 | Delara, R., Yi, J., Girardo, M., & Wasson, M. (2020). Perioperative outcomes of total vaginal hysterectomy in women with prior cesarean delivery. *Journal of Minimally Invasive Gynecology*, *27*(7), 1603-1609. | Unable to extract data | 27 |
| 1811 | El Bayoumy, R., Guirgis, R., & Guyer, C. (2011). Trans-abdominal plane (TAP) block analgesia for day-case laparoscopic gynaecological procedures: A prospective study: 14AP5-2. *European Journal of Anaesthesiology\| EJA*, *28*, 201. (Abstract) | Wrong source | 28 |
| 1842 | Ellinides, A., Manolopoulos, P. P., Hajymiri, M., Sergentanis, T. N., Trompoukis, P., & Ntourakis, D. (2021). Outpatient Hysterectomy vs Inpatient Hysterectomy: A Systematic Review and Meta-analysis, *The Journal of Minimally Invasive Gynecology*, <https://doi.org/10.1016/j.jmig.2021.06.012> (No peer-reviewed version found.) | Wrong source | 29 |
| 1851 | Ely, K., Stafflinger, J., & Goldberg, A. (2016). Efficacy of the Routine Postoperative Visit in Benign Gynecologic Ambulatory Procedures. *Journal of Minimally Invasive Gynecology*, *23*(7), S169-S170. (Video poster) | Wrong source | 30 |
| 2036 | Fountain, C. R., & Havrilesky, L. J. (2017). Promoting same-day discharge for gynecologic oncology patients in minimally invasive hysterectomy. *Journal of Minimally Invasive Gynecology*, *24*(6), 932-939. | Harmful incidents not reported | 31 |
| 2038 | Fowler, M. L., Delgado, S., Hendessi, P., Memmo, E., Iverson, R., White, K., & Noel, N. L. (2019). 1645 Same Day Discharge after Minimally Invasive Gynecologic Surgery at an Urban, Safety-Net Hospital. *Journal of Minimally Invasive Gynecology*, *26*(7), S127-S128. (Poster) | Wrong source | 32 |
| 2039 | Fowler, M. L., Pokuaa, I., Delgado, S., Hendessi, P., Memmo, E., Iverson, R. E., White, K., & Noel, N. L. (2022). Implementation of Same-Day Discharge in Minimally Invasive Gynecologic Surgery in a Safety-Net Hospital. *Indian Journal of Surgery*, *84*(6), 1245–1252. <https://doi.org/10.1007/s12262-021-03225-y> | Duplicate | 33 |
| 2093 | Gabarin, N., Sirotich, E., Liu, Y., Pai, M., Luketic, L., Arnold, D. M., & Zeller, M. P. (2021). Perioperative Anemia Management in Women Undergoing Gynecologic Procedures: A 10 Year Multisite Study. *Blood*, *138*, 752. (Abstract) | Wrong source | 34 |
| 2306 | Goravanchi, F., Rigby, D., Carson, S., French, K., Kowalski, A., Rebello, E., Kee, S., Lucci, A., Caudel, A., Kuerer, H. Outpatient breast and gynecological surgery is safe: Recent experience with 11,156 pateints at M. D. Anderson cancer center. (Poster) | Wrong source | 35 |
| 2340 | Gran Bruun, A., Svensen, K., Johansen, E., Halstensen, T., Gustavsson, A., & Leonardsen, A. L. (2023). A quantitative, multicentre, longitudinal study of patient experiences after gynaecological day surgery. *Nursing Open*, *10*(3), 1536–1544. <https://doi.org/10.1002/nop2.1403> | Eligibility could not be confirmed | 36 |
| 2442 | Gupta, S., Maghsoudlou, P., Ajao, M., Einarsson, J. I., & King, L. P. (2022). Very low rates of ureteral injury in laparoscopic hysterectomy performed by fellowship-trained minimally invasive gynecologic surgeons. *Journal of Minimally Invasive Gynecology*, *29*(9), 1099-1103. | Harmful incidents not reported | 37 |
| 2486 | Haight, P. J., Barrington, D. A., Graves, S. M., Piver, R. N., Baek, J., Ardizzone, M., Akinduro, J. A., Busho, A. C., Fadoju, D., Pandit, R., Stephens, R., Strowder, L. M., Tadepalli, S., VanNoy, B., Sriram, B., McLaughlin, E. M., Lightfoot, M. D. S., Bixel, K. L., Cohn, D. E., … Backes, F. J. (2023). Safety and feasibility of same-day discharge following minimally invasive hysterectomy in the morbidly obese patient population. *Gynecologic Oncology*, *170*, 203–209. <https://doi.org/10.1016/j.ygyno.2023.01.013> | Harmful incidents not reported | 38 |
| 2574 | Hart, S. T., Nelson, M., Kirshenbaum, E., Chen, Y., Mueller, E. R., & Gupta, G. (2020). Post-hospital syndrome predicts poor postoperative outcomes and increased cost following transvaginal midurethral sling placement. *International Urogynecology Journal*, *31*(7), 1417–1422. <https://doi.org/10.1007/s00192-019-04009-5> | Wrong surgeries | 39 |
| 2655 | Hickman, L., Ferrando, C., Goldman, H., Propst, K., Paraiso, M. F. Same-day discharge should be implemented after minimally invasive sacrocolpopexy. (Abstract) | Wrong source | 40 |
| 2656 | Hickman, L. C., Paraiso, M. F. R., Goldman, H. B., Propst, K., & Ferrando, C. A. (2021). Same-Day Discharge After Minimally Invasive Sacrocolpopexy Is Feasible, Safe, and Associated With High Patient Satisfaction. *Female pelvic medicine & reconstructive surgery*, *27*(8), e614–e619. <https://doi.org/10.1097/SPV.0000000000000998> | Unable to extract data | 41 |
| 2662 | Hilmy, M., Heaton, S., Urwin, G. Single incision mid urethral sling for urinary in continence: Small tape for a big problem | Wrong surgeries | 42 |
| 2782 | Hunter, J. D., Chambers, W. A., & Penny, K. I. (1998). Minor morbidity after day-case surgery. *Scottish medical journal*, *43*(2), 54-56. (Commentary) | Wrong source | 43 |
| 2955 | Jennings, A. J., Spencer, R. J., Medlin, E., Rice, L. W., & Uppal, S. (2015). Predictors of 30-day readmission and impact of same-day discharge in laparoscopic hysterectomy. *American Journal of Obstetrics and Gynecology*, *213*(3), 344.e1-344.e7. <https://doi.org/10.1016/j.ajog.2015.05.014> | Wrong population | 44 |
| 2960 | Jensen, L. B., Jeppesen, U., & Bor, P. (2022). Risk of deep vein thrombosis and pulmonary embolism after gynecological day surgery. *European Journal of Obstetrics & Gynecology and Reproductive Biology*, *270*, 1–5. <https://doi.org/10.1016/j.ejogrb.2021.12.027> | Unable to extract data | 45 |
| 2974 | Salas, B. J., Frontera, M. R., García, B. S., García, F. G. Á., Bernadó, A. J., & Albareda, J. A. (2020). Causes of unplanned admission after orthopaedic procedures in ambulatory surgery. *Revista Española de Cirugía Ortopédica y Traumatología (English Edition)*, *64*(1), 50-56. | Wrong population | 46 |
| 3015 | Jorgensen, E. M., Li, A., Modest, A. M., Leung, K., Simas, T. A. M., & Hur, H. C. (2018). Incidence of venous thromboembolism after different modes of gynecologic surgery. *Obstetrics & Gynecology*, *132*(5), 1275-1284. | Unable to extract data | 47 |
| 3123 | Katz, M. S., Gandhi, A., Valente, A., Richardson, D., & Holman, L. L. (2022). 43 Implementation of a standard same day discharge protocol for minimally invasive hysterectomies in the division of gynecologic oncology: a quality improvement project. Gynecologic Oncology Reports, 44, S21-S22. (Poster) | Wrong source | 48 |
| 3163 | Kepner, S., & Jones, R. (2022). Patient Safety Trends in 2021: An Analysis of 288,882 Serious Events and Incidents From the Nation’s Largest Event Reporting Database. *Patient Safety (Harrisburg, Pa. Online)*, *4*(2), 18–33. <https://doi.org/10.33940/data/2022.6.2> | Unable to extract data | 49 |
| 3179 | Keyes, G. R., Singer, R., Iverson, R. E., McGuire, M., Yates, J., Gold, A., & Thompson, D. (2004). Analysis of outpatient surgery center safety using an internet-based quality improvement and peer review program. *Plastic and reconstructive surgery*, *113*(6), 1760–1770. <https://doi.org/10.1097/01.prs.0000124743.75839.11> | Wrong population | 50 |
| 3207 | Kho, R. M., Chang, O. H., Hare, A., Schaffer, J., Hamner, J., Northington, G. M., Metcalfe, N. D., Iglesia, C. B., Zelivianskaia, A. S., Hur, H. C., Seaman, S., Mueller, M. G., Milad, M., Ascher-Walsh, C., Kossl, K., Rardin, C., Siddique, M., Murphy, M., & Heit, M. (2022). Surgical Outcomes in Benign Gynecologic Surgery Patients during the COVID-19 Pandemic (SOCOVID study). *Journal of minimally invasive gynecology*, *29*(2), 274–283.e1. <https://doi.org/10.1016/j.jmig.2021.08.011> | Unable to extract data | 51 |
| 3232 | Kim, E. K., Hong, C. X., & Harvie, H. S. (2022). Trends in Same-Day Discharge Rate After Minimally Invasive Sacrocolpopexy and Propensity Score-Matched Analysis of Postoperative Complication Rates Using the National Surgical Quality Improvement Program Database. *Female pelvic medicine & reconstructive surgery*, *28*(3), e22–e28. <https://doi.org/10.1097/SPV.0000000000001139> | Wrong population | 52 |
| 3569 | Lee, C. E., & Epp, A. (2018). Safety and Efficiency in a Canadian Outpatient Gynaecological Surgical Centre. *Journal of obstetrics and gynaecology Canada : JOGC = Journal d'obstetrique et gynecologie du Canada : JOGC*, *40*(4), 426–431. <https://doi.org/10.1016/j.jogc.2017.07.027> | Wrong population | 53 |
| 3576 | Lee, J., Aphinyanaphongs, Y., Curtin, J. P., Chern, J.-Y., Frey, M. K., & Boyd, L. R. (2016). The safety of same-day discharge after laparoscopic hysterectomy for endometrial cancer. *Gynecologic Oncology*, *142*(3), 508–513. https://doi.org/10.1016/j.ygyno.2016.06.010 | Wrong population | 54 |
| 3577 | Lee, J., Brodsky, A. L., Figueroa, M. A., Stamm, M. H., Huncke, T. W., Jain, S. K., ... & Pothuri, B. (2019). Optimizing gynecologic surgery for the morbidly obese patient with a surgical safety pathway. *Gynecologic Oncology*, *154*, 139. (Poster) | Wrong source | 55 |
| 3636 | Lennox, G. K., Peng, J., Murphy, K. J., Pulman, K. J., Sinasac, S. E., & Feigenberg, T. (2020). Same-Day Discharge Among Patients With Obesity Undergoing Laparoscopic Gynaecologic Oncology Surgery. *Journal of obstetrics and gynaecology Canada : JOGC = Journal d'obstetrique et gynecologie du Canada : JOGC*, *42*(8), 957–962. <https://doi.org/10.1016/j.jogc.2020.01.022> | Harmful incidents not reported | 56 |
| 3704 | Lightfoot, M. D. S., Felix, A. S., Bishop, E. E., Henderson, A. P., Vetter, M. H., Salani, R., O’Mallley, D. M., Bixel, K. L., Cohn, D. E., Fowler, J. M., & Backes, F. J. (2022). Who will be readmitted? Evaluation of the laparoscopic hysterectomy readmission score in a gynecologic oncology population undergoing robotic-assisted hysterectomy. *Gynecologic Oncology*, *164*(3), 628–638. <https://doi.org/10.1016/j.ygyno.2021.12.010> | Unable to extract data | 57 |
| 3787 | Lo, S., Stuenkel, D. L., & Rodriguez, L. (2009). The Impact of Diagnosis-Specific Discharge Instructions on Patient Satisfaction. *Journal of Perianesthesia Nursing*, *24*(3), 156–162. <https://doi.org/10.1016/j.jopan.2009.03.004> | Unable to extract data | 58 |
| 3823 | López-Torres López, J., Cifuentes García, B., Fernández Ruipérez, L., Rodeles Criado, A., Alcántara Noalles, M. J., Peiró García, R., & Argente Navarro, P. (2021). Predictive Factors of Admission in Outpatient Laparoscopic Surgery. Cirugia Española (English Ed.), 99(2), 140–146. <https://doi.org/10.1016/j.ciresp.2020.04.023> | Unable to extract data | 59 |
| 3852 | Luchristt, D., Kenton, K. S., Bretschneider, C. E. Historical and Forecasted Changes in Utilization of Same-day Discharge after Minimally Invasive Hysterectomy (Poster/abstract) | Wrong source | 60 |
| 3902 | MacKoul, P., Danilyants, N., Touchan, F., van der Does, L. Q., Haworth, L. R., & Kazi, N. (2020). Laparoscopic-assisted myomectomy with uterine artery occlusion at a freestanding ambulatory surgery center: a case series. *Gynecological Surgery*, *17*(1). <https://doi.org/10.1186/s10397-020-01075-2> | Harmful incidents not reported | 61 |
| 3911 | Madden, N., Frey, M. K., Joo, L., Lee, J., Musselman, K., Chern, J. Y., Blank, S. V., & Pothuri, B. (2019). Safety of robotic-assisted gynecologic surgery and early hospital discharge in elderly patients. American Journal of Obstetrics and Gynecology, 220(3), 253.e1-253.e7. <https://doi.org/10.1016/j.ajog.2018.12.014> | Harmful incidents not reported | 62 |
| 3916 | Costa, E. A., Moreira, L. L., & Gusmão, M. E. (2019). Incidence of infection of surgical site in hospital day: cohort of 74,213 patients monitored. *Rev Sobecc*, *24*(4), 211-6. | Harmful incidents not reported | 63 |
| 3940 | Majholm, B., Esbensen, B. A., Thomsen, T., Engbæk, J., & Møller, A. M. (2012). Partners’ experiences of the postdischarge period after day surgery - a qualitative study. *Journal of Clinical Nursing*, *21*(17–18), 2518–2527. <https://doi.org/10.1111/j.1365-2702.2012.04116.x> | Wrong surgeries | 64 |
| 4046 | Martins, M., Vaz, I., Barbosa, H., Coroa, M., Brás, A., & Amaro, L. (2023). Individualized Care and Follow-Up in Outpatient Surgery: A Pilot Study. *Curēus (Palo Alto, CA)*, *15*(1), e33698–e33698. <https://doi.org/10.7759/cureus.33698> | Unable to extract data | 65 |
| 4066 | Matern, T., Kang, E., & Lim, P. C. (2020). Factors in the feasibility and safety of outpatient robotic-assisted hysterectomy for endometrial or cervical carcinoma. *Gynecologic Oncology*, *157*(2), 482–486. <https://doi.org/10.1016/j.ygyno.2020.01.028> | Unable to extract data | 66 |
| 4126 | McGrath, B., & Chung, F. (2003). Postoperative recovery and discharge. *Anesthesiology Clinics of North America*, *21*(2), 367-386. | Harmful incidents not reported | 67 |
| 4132 | McIsaac, D. I., Bryson, G. L., & van Walraven, C. (2015). Impact of ambulatory surgery day of the week on postoperative outcomes: a population-based cohort study. *Canadian Journal of Anesthesia*, *62*(8), 857–865. <https://doi.org/10.1007/s12630-015-0408-x> | Wrong population | 68 |
| 4201 | Meshkat, B., Soon, Y. H., Gethin, G., Cowman, S., Wiley, M., Brick, A., Clarke, E., Walsh, T. N. Adverse events post day surgery; A survey from an Irish teaching hospital. (Abstract) | Wrong source | 69 |
| 4377 | Moulton, L. J., Jernigan, A. M., & Michener, C. M. (2017). Postoperative Outcomes after Single-port Laparoscopic Removal of Adnexal Masses in Patients Referred to Gynecologic Oncology at a Large Academic Center. *Journal of minimally invasive gynecology*, *24*(7), 1136–1144. <https://doi.org/10.1016/j.jmig.2017.06.023> | Wrong population | 70 |
| 4400 | Mull, H. J., Itani, K. M. F., Charns, M. P., Pizer, S. D., Rivard, P. E., Hawn, M. T., & Rosen, A. K. (2018). The Nature and Severity of Adverse Events in Select Outpatient Surgical Procedures in the Veterans Health Administration. *Quality management in health care*, *27*(3), 136–144. <https://doi.org/10.1097/QMH.0000000000000177> | Unable to extract data | 71 |
| 4401 | Mull, H. J., Itani, K. M. F., Pizer, S. D., Charns, M. P., Rivard, P. E., McIntosh, N., Hawn, M. T., & Rosen, A. K. (2018). Development of an Adverse Event Surveillance Model for Outpatient Surgery in the Veterans Health Administration. *Health services research*, *53*(6), 4507–4528. <https://doi.org/10.1111/1475-6773.13037> | Unable to extract data | 72 |
| 4405 | Mull, H. J., Rosen, A. K., O’Brien, W. J., McIntosh, N., Legler, A., Hawn, M. T., Itani, K. M. F., & Pizer, S. D. (2018). Factors Associated with Hospital Admission after Outpatient Surgery in the Veterans Health Administration. *Health Services Research*, *53*(5), 3855–3880. <https://doi.org/10.1111/1475-6773.12826> | Wrong population | 73 |
| 4524 | Nensi, A., Coll-Black, M., Leyland, N., & Sobel, M. L. (2018). Implementation of a Same-Day Discharge Protocol Following Total Laparoscopic Hysterectomy. *Journal of obstetrics and gynaecology Canada: JOGC = Journal d'obstetrique et gynecologie du Canada : JOGC*, *40*(1), 29–35. <https://doi.org/10.1016/j.jogc.2017.05.035> | Harmful incidents not reported | 74 |
| 4670 | Ojo, E. O., Ihezue, C. H., Sule, A. Z., Dakum, N. K., & Misauno, M. A. (2008). The safety of day case surgery in a developing country. *Journal of One Day Surgery*, *18*(1), 13. | Wrong population | 75 |
| 4703 | Orlando, M. S., Yao, M., Chang, O. H., Shippey, E., Bosko, T., Cadish, L., Falcone, T., & Kho, R. M. (2022). Perioperative outcomes in a nationwide sample of patients undergoing surgical treatment of ovarian endometriomas. *Fertility and Sterility*, *117*(2), 444–453. <https://doi.org/10.1016/j.fertnstert.2021.10.008> | Wrong population | 76 |
| 4727 | Owens, P. L., Barrett, M. L., Raetzman, S., Maggard-Gibbons, M., & Steiner, C. A. (2014). Surgical Site Infections Following Ambulatory Surgery Procedures. *JAMA : The Journal of the American Medical Association*, *311*(7), 709–716. <https://doi.org/10.1001/jama.2014.4> | Wrong population | 77 |
| 4862 | Peacock, L. M., Thomassee, M. E., Williams, V. L., & Young, A. E. (2015). Transition to Office-based Obstetric and Gynecologic Procedures: Safety, Technical, and Financial Considerations. *Clinical obstetrics and gynecology*, *58*(2), 418–433. <https://doi.org/10.1097/GRF.0000000000000100> | Wrong population | 78 |
| 4881 | Patel, S. (2007). A prospective observational study of the safety and acceptability of vaginal hysterectomy performed in a 24‐hour day case surgery setting. *BJOG : An International Journal of Obstetrics and Gynaecology*, *114*(8), 1045–1045. <https://doi.org/10.1111/j.1471-0528.2007.01402.x> | Wrong population | 79 |
| 4905 | Perry, C. P., Presthus, J., & Nieves, A. (2005). Laparoscopic uterine suspension for pain relief: a multicenter study. *The Journal of reproductive medicine*, *50*(8), 567–570. | Harmful incidents not reported | 80 |
| 4919 | Pham, A., Kung, R. C., Wong, H. M., Liu, G. Y., Kroft, J., Bodley, J. L., & Lee, P. E. (2012). Same-Day Discharge Versus Overnight Stay after Laparoscopic Hysterectomy: A Prospective Assessment of Patient Safety and Patient Satisfaction. *Journal of Minimally Invasive Gynecology*, *19*(6), S153. (Abstract) | Wrong source | 81 |
| 4942 | Pierce, H., Forshaw, C., Godfrey, M. A. L., Craig, N. V., Stezaker, S., & Gardner, F. (2022). 2022-RA-1366-ESGO Implementation of day case minimal access hysterectomy. *International Journal of Gynecological Cancer*, *32*(Suppl 2), A461-A461. (Abstract) | Wrong source | 82 |
| 5022 | Praiss, A. M., Chen, L., St Clair, C. M., Tergas, A. I., Khoury-Collado, F., Hou, J. Y., Ananth, C. V., Neugut, A. I., Hershman, D. L., & Wright, J. D. (2019). Safety of same-day discharge for minimally invasive hysterectomy for endometrial cancer. *American Journal of Obstetrics and Gynecology*, *221*(3), 239.e1-239.e11. <https://doi.org/10.1016/j.ajog.2019.05.003> | Wrong population | 83 |
| 5065 | Qi, M., Lopa, S., Adambekov, S., Harris, J. A., Mansuria, S., Edwards, R. P., & Linkov, F. (2021). Same-day discharge after minimal invasive hysterectomy: Applications for improved value of care. *European Journal of Obstetrics & Gynecology and Reproductive Biology*, *259*, 140–145. <https://doi.org/10.1016/j.ejogrb.2021.02.020> | Harmful incidents not reported | 84 |
| 5144 | Rangel-Frausto, M. S., Martinez-Abaroa, C., Ponce De León, S. Ambulatory surgical wound surveillance: Beyond infections. (Abstract) | Wrong source | 85 |
| 5297 | Robaux, S., Bouaziz, H., Cornet, C., Boivin, J. M., Lefèvre, N., & Laxenaire, M. C. (2002). Acute Postoperative Pain Management at Home After Ambulatory Surgery: A French Pilot Survey of General Practitioners’ Views. *Anesthesia and Analgesia*, *95*(5), 1258–1262. <https://doi.org/10.1097/00000539-200211000-00029> | Harmful incidents not reported | 86 |
| 5373 | Rosenfield, R. B., & Dow, R. (2010). The completely outpatient hysterectomy–a case series of 500 consecutive patients with same day discharge home. *Journal of Minimally Invasive Gynecology*, *17*(6), S56-S57. (Abstract) | Wrong source | 87 |
| 5390 | Rothenberg, K. A., Stern, J. R., George, E. L., Trickey, A. W., Morris, A. M., Hall, D. E., Johanning, J. M., Hawn, M. T., & Arya, S. (2019). Association of frailty and postoperative complications with unplanned readmissions after elective outpatient surgery. JAMA Network Open, 2(5), e194330–e194330. <https://doi.org/10.1001/jamanetworkopen.2019.4330> | Harmful incidents not reported | 88 |
| 5496 | Salwei, M. E., Anders, S., Slagle, J. M., Whitney, G., Lorinc, A., Morley, S., Pasley, J., Declercq, J., Shotwell, M. S., & Weinger, M. B. (2023). Understanding Patient and Clinician Reported Nonroutine Events in Ambulatory Surgery. *Journal of Patient Safety*, *19*(2), E38–E45. <https://doi.org/10.1097/PTS.0000000000001089> | Not ambulatory surgery | 89 |
| 5513 | Sanabria, D., Rodriguez, J., Pecci, P., Ardila, E., & Pareja, R. (2020). Same-Day Discharge in Minimally Invasive Surgery Performed by Gynecologic Oncologists: A Review of Patient Selection. *Journal of minimally invasive gynecology*, *27*(4), 816–825. <https://doi.org/10.1016/j.jmig.2019.10.023> | Harmful incidents not reported | 90 |
| 5583 | Schiavone, Maria B., MD, Herzog, Thomas J., MD, Ananth, Cande V., PhD, MPH, Wilde, Elizabeth T., PhD, Lewin, Sharyn N., MD, Burke, William M., MD, Lu, Yu-Shiang, MS, Neugut, Alfred I., MD, PhD, Hershman, Dawn L., MD, & Wright, Jason D., MD. (2012). Feasibility and economic impact of same-day discharge for women who undergo laparoscopic hysterectomy. *American Journal of Obstetrics and Gynecology*, *207*(5), 382.e1-382.e9. <https://doi.org/10.1016/j.ajog.2012.09.014> | Wrong population | 91 |
| 5736 | Sharma, A. (2018). Day-case total laparoscopic hysterectomy (TLH): an audit of safety and practicality of procedure at Sunderland royal hospital (SRH). *BJOG : An International Journal of Obstetrics and Gynaecology.*, *125*. (Poster) | Wrong source | 92 |
| 5737 | (Duplicate of Ref ID 5736.) | Duplicate | 93 |
| 5799 | Siebert, D., Giraudet, G., Collinet, P., Gonzalez Estevez, M., Cosson, M., & Rubod, C. (2022). Risk factors for immediate failure of outpatient surgery in gynecologic surgery. *International Journal of Gynecology and Obstetrics*, *159*(2), 592–599. <https://doi.org/10.1002/ijgo.14220> | Unable to extract data | 94 |
| 5859 | Singletary D. (2016). Evaluation of the safety and efficacy of same-day discharge following outpatient surgery in a US hospital. *Nursing management (Harrow, London, England : 1994)*, *23*(4), 34–38. <https://doi.org/10.7748/nm.2016.e1410> | Harmful incidents not reported | 95 |
| 5879 | Slowey, M. J., & Coddington, C. C. (1998). Microsurgical Tubal Anastomoses Performed as an Outpatient Procedure by Minilaparotomy are Less Expensive and as Safe as Those Performed as an Inpatient Procedure. *Fertility and Sterility*, *69*(3), 492–495. <https://doi.org/10.1016/S0015-0282(97)00549-9> | Harmful incidents not reported | 96 |
| 5937 | Son, J., Tran, T., Yao, M., & Michener, C. M. (2022). Factors Associated With Unplanned Admission in Patients Intended for Same Day Discharge After Minimally Invasive Hysterectomy for Endometrial Cancer. *Surgical Innovation*, *29*(3), 336–342. <https://doi.org/10.1177/15533506211041882> | Harmful incidents not reported | 97 |
| 6344 | Trister, R., Jacobson, M., Nguyen, P., Sobel, M., Allen, L., Narod, S. A., & Kotsopoulos, J. (2021). Patient reported experiences following laparoscopic prophylactic bilateral salpingo-oophorectomy or salpingectomy in an ambulatory care hospital. *Familial Cancer*, *20*(2), 103–110. <https://doi.org/10.1007/s10689-020-00208-y> | Unable to extract data | 98 |
| 6385 | Tutoveanu, G. (2022). Daycase laparoscopic hysterectomy: A safe, cost effective option in a district hospital. *BJOG : An International Journal of Obstetrics and*  *Gynaecology.*, *129*. (Abstract) | Wrong source | 99 |
| 6396 | Tyson, M. D., & Wolter, C. E. (2015). A comparison of 30‐day surgical outcomes for minimally invasive and open sacrocolpopexy. *Neurourology and Urodynamics*, *34*(2), 151-155. | Wrong population | 100 |
| 6523 | Vetuz, G. (2016). Patient experience and outcome following day case surgery. *Anaesthesia.*, *71*. (Abstract) | Wrong source | 101 |
| 6581 | Wainger, J. J., Yazdy, G. M., & Handa, V. L. (2022). Abdominal hysterectomy and high frailty score are associated with complications among older patients. *International journal of gynaecology and obstetrics: the official organ of the International Federation of Gynaecology and Obstetrics*, *158*(3), 544–550. <https://doi.org/10.1002/ijgo.14029> | Harmful incidents not reported | 102 |
| 6610 | Walsh, M. T. (2021). Discharging select patients without an escort after ambulatory anesthesia: identifying return to baseline function. *Current Opinion in Anesthesiology*, *34*(6), 703-708. | Wrong source | 103 |
| 6619 | Berger, A. A., Tan-Kim, J., & Menefee, S. A. (2021). Utilizing Outpatient Pelvic Reconstructive Surgery in the Era of the COVID-19 Pandemic. *Female pelvic medicine & reconstructive surgery*, *27*(12), 735–739. <https://doi.org/10.1097/SPV.0000000000001044> | Harmful incidents not reported | 104 |
| 7028 | Zivanovic, O., Chen, L. Y., Vickers, A., Straubhar, A., Baser, R., Veith, M., Aiken, N., Carter, J., Curran, K., Simon, B., Mueller, J., Jewell, E., Chi, D. S., Sonoda, Y., Abu-Rustum, N. R., & Leitao, M. M. (2020). Electronic patient-reported symptom monitoring in patients recovering from ambulatory minimally invasive gynecologic surgery: A prospective pilot study. *Gynecologic Oncology*, *159*(1), 187–194. <https://doi.org/10.1016/j.ygyno.2020.07.004> | Wrong population | 105 |
| 7029 | Zivanovic, O., Veith, M. W., Baser, R. E., Jewell, E., Chen, L. Y., Brown, C. L., ... & Leitao, M. M. (2017). Automated patient-reported symptom data capture, tracking, and intervention during recovery from ambulatory gynecologic cancer surgery: A prospective study. *Gynecologic Oncology*, *145*, 47. (Poster) | Wrong source | 106 |
| 7030 | Zivanovic, O., Vickers, A. J., Baser, R. E., Curran, K. G., Veith, M. W., Abu-Rustum, N. R., ... & Leitao, M. M. (2019). Electronic symptom monitoring with patient-reported outcomes in patients recovering from ambulatory minimally invasive gynecologic cancer surgery: A prospective pilot study. *Gynecologic Oncology*, *154*, 164. (Poster) | Wrong source | 107 |
| 7287 | Haight, P. J., Barrington, D. A., Graves, S. M., Piver, R. N., Baek, J., Ardizzone, M., Akinduro, J. A., Busho, A. C., Fadoju, D., Pandit, R., Stephens, R., Strowder, L. M., Tadepalli, S., VanNoy, B., Sriram, B., McLaughlin, E. M., Lightfoot, M. D. S., Bixel, K. L., Cohn, D. E., … Backes, F. J. (2023). Safety and feasibility of same-day discharge following minimally invasive hysterectomy in the morbidly obese patient population. *Gynecologic Oncology*, *170*, 203–209. <https://doi.org/10.1016/j.ygyno.2023.01.013> | Duplicate | 108 |
| 7663 | Stork, A., King, L. A., Jaraki, D., Pruszynski, J., Florian-Rodriguez, M. Factors associated with failed same-day discharge in patients undergoing urogynecologic surgery. (Poster) | Wrong source | 109 |
| 7802 | Bai, L., Huang, Y., Huang, C., & Tan, X. (2024). Impact of Comprehensive Preoperative Assessments on Gynecological Ambulatory Surgery Outcomes in a Chinese Hospital. *Medical Science Monitor*, *30*, e945771. <https://doi.org/10.12659/MSM.945771> | Wrong population | 110 |
| 7817 | Bekkers, I. P. W., Henschen, R., Smeets, N. A. C., van Vliet, H., Damoiseaux, A., & Wassen, M. M. L. H. (2024). *Vaginal assisted NOTES hysterectomy in The Netherlands : a prospective cohort study*. | Unable to extract data | 111 |
| 7906 | Cracchiolo, J. R., Tin, A. L., Assel, M., McCready, T. M., Stabile, C., Simon, B., Carlsson, S. V., Vickers, A. J., & Laudone, V. (2024). Electronic Patient-Reported Symptoms After Ambulatory Cancer Surgery. *JAMA Surgery*, *159*(5), 554–561. <https://doi.org/10.1001/jamasurg.2024.0133> | Wrong population | 112 |
| 7921 | Dedden, S. J., Maas, J. W. M., Smeets, N. A. C., Hamont, D., Groenman, F. A., Lim, A. C., Vliet, H. A. A. M., Steeg, J. W., Leemans, J. C., Meijer, P., Kuijk, S. M. J., Huirne, J. A. F., Bongers, M. Y., & Geomini, P. M. A. J. (2024). Same‐day discharge after laparoscopic hysterectomy for benign/premalignant disease: A multicentre randomised controlled trial. *BJOG : An International Journal of Obstetrics and Gynaecology*, *131*(13), 1762–1770. <https://doi.org/10.1111/1471-0528.17911> | Unable to extract data | 113 |
| 8403 | Wang, X.-L., Dai, L.-L., Li, Y.-N., Zhang, J.-W., Qu, M.-C., Zhou, Y.-Y., & Xing, N. (2024). Comparing Remimazolam and Propofol for Postoperative Anesthesia Satisfaction in Outpatient Gynecological Surgery: A Randomized Clinical Trial. *Drug Design, Development and Therapy*, *18*, 4615–4627. <https://doi.org/10.2147/DDDT.S483029> | Wrong surgeries | 114 |
| 8410 | Wei, Y., Zhu, M., Man, Y., Xiao, H., Dong, G., Shi, X., & Ji, F. (2024). Clinical Study of Flumazenil Antagonizing Remimazolam on Nausea and Vomiting After Gynecologic Day Surgery. *Drug Design, Development and Therapy*, *18*, 631–638. <https://doi.org/10.2147/DDDT.S444313> | Wrong population | 115 |
| 8505 | Fuller, R. L., Hughes, J. S., Young, S. D., Fogerty, R., Wadhwa, S., Casey, D., ... & Chen, Y. (2025). Complications of Ambulatory Procedures: Prevalence and Hospital Outpatient Department Variation. *American Journal of Medical Quality*, *40*(2), 44-52. | Wrong population | 116 |
| 8547 | Sekar, H., Thiyagalingam, S., Swann, P., Karavolos, S., & Yoong, W. (2025). Enhanced recovery revisited: what day case hysterectomies can learn from Team GB elite athletes. *The Obstetrician & Gynaecologist*, *27*(1), 68–74. <https://doi.org/10.1111/tog.1295> | Wrong source | 117 |
| RECORDS IDENTIFIED FROM BACKWARD CHAINING REFERENCE LISTS OF INCLUDED ARTICLES | | | |
| Bibliographic information | | **Reason for exclusion** | **Number of records** |
| Keil, D. S., Schiff, L. D., Carey, E. T., Moulder, J. K., Goetzinger, A. M., Patidar, S. M., Hance, L. M., Kolarczyk, L. M., Isaak, R. S., Strassle, P. D., & Schoenherr, J. W. (2019). Predictors of Admission After the Implementation of an Enhanced Recovery After Surgery Pathway for Minimally Invasive Gynecologic Surgery. *Anesthesia and Analgesia*, *129*(3), 776. <https://doi.org/10.1213/ANE.0000000000003339> | | Unable to extract data | 1 |
| Dedden, S. J., Geomini, P. M., Huirne, J. A., & Bongers, M. Y. (2017). Vaginal and Laparoscopic hysterectomy as an outpatient procedure: A systematic review. *European Journal of Obstetrics & Gynecology and Reproductive Biology*, *216*, 212-223. | | Unable to extract data | 2 |
| Qi, M., Lopa, S., Adambekov, S., Harris, J. A., Mansuria, S., Edwards, R. P., & Linkov, F. (2021). Same-Day Discharge After Minimal Invasive Hysterectomy: Applications for Improved Value of Care. *European Journal of Obstetrics & Gynecology and Reproductive Biology*, *259*, 140–145. <https://doi.org/10.1016/j.ejogrb.2021.02.020> | | Harmful incidents not reported | 3 |
| Matern, T., Kang, E., & Lim, P. C. (2020). Factors in the feasibility and safety of outpatient robotic-assisted hysterectomy for endometrial or cervical carcinoma. *Gynecologic Oncology*, *157*(2), 482–486. <https://doi.org/10.1016/j.ygyno.2020.01.028> | | Unable to extract data | 4 |
| Nahas, S., Feigenberg, T., & Park, S. (2016). Feasibility and safety of same-day discharge after minimally invasive hysterectomy in gynecologic oncology: A systematic review of the literature. *Gynecologic Oncology*, *143*(2), 439–442. <https://doi.org/10.1016/j.ygyno.2016.07.113> | | Harmful incidents not reported | 5 |
| Korsholm, M., Mogensen, O., Jeppesen, M. M., Lysdal, V. K., Traen, K., & Jensen, P. T. (2017). Systematic review of same‐day discharge after minimally invasive hysterectomy. *INTERNATIONAL JOURNAL OF GYNECOLOGY & OBSTETRICS*, *136*(2), 128–137. <https://doi.org/10.1002/ijgo.12023> | | Harmful incidents not reported | 6 |
| MacKoul, P., Danilyants, N., Baxi, R., van der Does, L., & Haworth, L. (2019). Laparoscopic Hysterectomy Outcomes: Hospital vs Ambulatory Surgery Center. *Journal of the Society of Laparoendoscopic Surgeons*, *23*(1), e2018.00076. <https://doi.org/10.4293/JSLS.2018.00076> | | Unable to extract data | 7 |
| Keller, V., Rambeaud, C., Binelli, C., Gombaud, G., Agostini, A., & Villefranque, V. (2017). Feasibility of sacrocolpopexy by outpatient laparoscopic surgery. *Journal of Gynecology Obstetrics and Human Reproduction*, *46*(10), 727–730. <https://doi.org/10.1016/j.jogoh.2017.10.003> | | Unable to extract data | 8 |
| Alas, A. N., Espaillat, L., Plowright, L., Aguilar, V., & Davila, G. W. (2016). Same-day surgery for pelvic organ prolapse and urinary incontinence: Assessing satisfaction and morbidity. Perioperative Care and Operating Room Management, 5, 20–26. <https://doi.org/10.1016/j.pcorm.2016.12.003> | | Wrong surgeries | 9 |
| Carter-Brooks, C. M., Du, A. L., Ruppert, K. M., Romanova, A. L., & Zyczynski, H. M. (2018). Implementation of a urogynecology-specific enhanced recovery after surgery (ERAS) pathway. American Journal of Obstetrics and Gynecology, 219(5), 495.e1–495.e10. <https://doi.org/10.1016/j.ajog.2018.06.009> | | Unable to extract data | 10 |
| Alperin, M., Kivnick, S., & Poon, K. Y. T. (2012). Outpatient laparoscopic hysterectomy for large uteri. *Journal of Minimally Invasive Gynecology*, *19*(6), 689-694. | | Unable to extract data | 11 |
| De Lapasse, C., Rabischong, B., Bolandard, F., Canis, M., Botchorischvili, R., Jardon, K., & Mage, G. (2008). Total laparoscopic hysterectomy and early discharge: satisfaction and feasibility study. *Journal of Minimally Invasive Gynecology*, *15*(1), 20-25. | | Not ambulatory surgery | 12 |
| Lassen, P. D., Moeller-Larsen, H., & De Nully, P. (2012). Same-day discharge after laparoscopic hysterectomy. *Acta Obstetricia et Gynecologica Scandinavica*, *91*(11), 1339–1341. <https://doi.org/10.1111/j.1600-0412.2012.01535.x> | | Unable to extract data | 13 |
| Lieng, M., Istre, O., Langebrekke, A., Jungersen, M., & Busund, B. (2005). Outpatient laparoscopic supracervical hysterectomy with assistance of the lap loop. *Journal of minimally invasive gynecology*, *12*(3), 290-294. | | Study on medical device | 14 |
| Morrison Jr, J. E., & Jacobs, V. R. (2004). Outpatient laparoscopic hysterectomy in a rural ambulatory surgery center. *The Journal of the American Association of Gynecologic Laparoscopists*, *11*(3), 359-364. | | Harmful incidents not reported | 15 |
| Thiel, J., & Gamelin, A. (2003). Outpatient total laparoscopic hysterectomy. *The Journal of the American Association of Gynecologic Laparoscopists*, *10*(4), 481-483. | | Unable to extract data | 16 |
| Kisby, C. K., Polin, M. R., Visco, A. G., & Siddiqui, N. Y. (2019). Same-day discharge after robotic-assisted sacrocolpopexy. *Urogynecology*, *25*(5), 337-341. | | Harmful incidents not reported | 17 |
| Robison, E. H., Smith, P. E., Pandya, L. K., Nekkanti, S., Hundley, A. F., & Hudson, C. O. (2022). Readmissions and perioperative outcomes for same-day versus next-day discharge after prolapse surgery. International Urogynecology Journal, 33(7), 1897–1905. <https://doi.org/10.1007/s00192-021-04799-7> | | Wrong surgeries | 18 |
| Zillioux, J., Werneburg, G. T., & Goldman, H. B. (2021). Same‐day discharge across FPMRS surgical cases is safe and feasible: A 10‐year single‐surgeon experience. Neurourology and Urodynamics, 40(7), 1754–1760. <https://doi.org/10.1002/nau.24739> | | Wrong surgeries | 19 |
| Evans, S., Myers, E. M., & Vilasagar, S. (2019). Patient perceptions of same-day discharge after minimally invasive gynecologic and pelvic reconstructive surgery. American Journal of Obstetrics and Gynecology, 221(6), 621.e1–621.e7. <https://doi.org/10.1016/j.ajog.2019.06.046> | | Harmful incidents not reported | 20 |
| Praiss, A. M., Chen, L., St Clair, C. M., Tergas, A. I., Khoury-Collado, F., Hou, J. Y., Ananth, C. V., Neugut, A. I., Hershman, D. L., & Wright, J. D. (2019). Safety of same-day discharge for minimally invasive hysterectomy for endometrial cancer. American Journal of Obstetrics and Gynecology, 221(3), 239.e1–239.e11. <https://doi.org/10.1016/j.ajog.2019.05.003> | | Duplicate | 21 |
| Levy, B. S., Luciano, D. E., & Emery, L. L. (2005). Outpatient vaginal hysterectomy is safe for patients and reduces institutional cost. *Journal of minimally invasive gynecology*, *12*(6), 494-501. | | Unable to extract data | 22 |
| Taylor, R. H. (1994). Outpatient laparoscopic hysterectomy with discharge in 4 to 6 hours. *The Journal of the American Association of Gynecologic Laparoscopists*, *1*(4, Part 2), S35-S35. | | Wrong source | 23 |
| Kisic-Trope, J., Qvigstad, E., & Ballard, K. (2011). A randomized trial of day-case vs inpatient laparoscopic supracervical hysterectomy. American journal of obstetrics and gynecology, 204(4), 307-e1. | | Harmful incidents not reported | 24 |
| Doll, K. M., Dusetzina, S. B., & Robinson, W. (2016). Trends in inpatient and outpatient hysterectomy and oophorectomy rates among commercially insured women in the United States, 2000-2014. *JAMA surgery*, *151*(9), 876-877. | | Wrong source | 25 |
| Khavanin, N., Mlodinow, A., Milad, M. P., Bilimoria, K. Y., & Kim, J. Y. (2013). Comparison of perioperative outcomes in outpatient and inpatient laparoscopic hysterectomy. *Journal of Minimally Invasive Gynecology*, *20*(5), 604-610. | | Wrong source | 26 |
