# Supplementary material for: Harmful incidents following gynaecological ambulatory surgery: A scoping review
Source: Int J Nurs Stud Adv. 2026 Jan 7;10:100487. doi: 10.1016/j.ijnsa.2026.100487 (PMC12969120; doi:10.1016/j.ijnsa.2026.100487)
Supplement: Supplementary file 5 [file mmc5.docx]

**Supplementary material 4: Data extraction form**

| DATA EXTRACTION FORM | |
| --- | --- |
| Characteristics of the included source of evidence | |
| Authors: |  |
| Title: |  |
| Year: |  |
| Country: |  |
| Journal: |  |
| Funding: |  |
| Methodology | |
| Design and methods:  (Study design, date and total duration of study, number of study centres and location, study setting, and withdrawals.) |  |
| Objective(s) of the study: |  |
| Outcome measures:  (Primary and secondary outcomes specified and collected, and time points reported.) |  |
| Ethical considerations: |  |
| Participants | |
| Types of ambulatory surgery: |  |
| Methods of surgery:  (Laparoscopy, vaginal, etc.) |  |
| Type of anaesthesia: |  |
| Inclusion and exclusion criteria: |  |
| Sample size: |  |
| Time of data collection:  (Postoperative day/time after surgery) |  |
| Demographic details:  (Including mean age and variance/age range, ASA-score and BMI.) |  |
| Concept | |
| The types of harmful incidents reported after ambulatory surgery and their occurrence |  |
| Comments | |
|  | |
